# Enhanced aqueous formation and neutralization of fine atmospheric particles driven by extreme cold

**DOI:** 10.1126/sciadv.ado4373

**Published:** 2024-09-04

**Authors:** James R. Campbell, Michael Battaglia, Kayane K. Dingilian, Meeta Cesler-Maloney, William R. Simpson, Ellis S. Robinson, Peter F. DeCarlo, Brice Temime-Roussel, Barbara D’Anna, Andrew L. Holen, Judy Wu, Kerri A. Pratt, Jack E. Dibb, Athanasios Nenes, Rodney J. Weber, Jingqiu Mao

**Affiliations:** ^1^ Geophysical Institute and Department of Chemistry and Biochemistry, University of Alaska Fairbanks, Fairbanks, AK 99775, USA.; ^2^ School of Earth and Atmospheric Sciences, Georgia Institute of Technology, Atlanta, GA 30332, USA.; ^3^ Department of Environmental Health and Engineering, John Hopkins University, Baltimore, MD 21218, USA.; ^4^ Aix Marseille Univ, CNRS, LCE, Marseille, France.; ^5^ Department of Chemistry, University of Michigan, Ann Arbor, MI 48109, USA.; ^6^ Institute for the Study of Earth, Oceans, and Space, University of New Hampshire, Durham, NH 03824, USA.; ^7^ Laboratory of Atmospheric Processes and their Impacts, School of Architecture, Civil and Environmental Engineering, École Polytechnique Fédérale de Lausanne, Lausanne 1015, Switzerland.; ^8^ Center for the Study of Air Quality and Climate Change, Institute of Chemical Engineering Sciences, Foundation for Research and Technology Hellas, Patras 26504, Greece.

## Abstract

The prevailing view for aqueous secondary aerosol formation is that it occurs in clouds and fogs, owing to the large liquid water content compared to minute levels in fine particles. Our research indicates that this view may need reevaluation due to enhancements in aqueous reactions in highly concentrated small particles. Here, we show that low temperature can play a role through a unique effect on particle pH that can substantially modulate secondary aerosol formation. Marked increases in hydroxymethanesulfonate observed under extreme cold in Fairbanks, Alaska, demonstrate the effect. These findings provide insight on aqueous chemistry in fine particles under cold conditions expanding possible regions of secondary aerosol formation that are pH dependent beyond conditions of high liquid water.

## Teaser

Aqueous aerosol formation is believed to occur in clouds/fogs; measurements expand possible regions of pH-dependent formation.

## INTRODUCTION

Air pollution in populated regions is often characterized as emissions of pollutants like sulfur dioxide (SO_2_), nitrogen oxides (NO_X_), volatile organic compounds, and particulate matter (PM). Emissions of these primary pollutants into a shallow boundary layer under low sunlight and cold temperatures, typical of wintertime conditions at higher latitudes, lead to severe pollution episodes, characterized by very high PM_2.5_ (aerodynamic diameter of <2.5 μm) levels (*1*). The role of secondary aerosol chemistry as a source of PM_2.5_ under wintertime conditions remains unclear, as secondary aerosols are typically associated with higher temperatures and light levels. Low temperatures, however, may facilitate PM formation through decreased volatility of gas-phase species and through effects on chemical kinetics, and the nitrate radical can rapidly oxidize primary emissions even under dark wintertime conditions (*2*). Fairbanks winter provides an ideal case to examine secondary aerosol formation under very cold and dark conditions. Fairbanks, Alaska (latitude 64.84°N), is a subarctic city with high wintertime concentrations of PM_2.5_ and wintertime temperatures often between −20° and −30°C, with episodes below −40°C. In this temperature range, and with moderately high relative humidity (RH; 70 to 80%), we expect particulate ammonium sulfate to be a supercooled solution (*3*) and that ambient particles with additional solutes, such as organic species, likely effloresce at even lower temperatures due to the plasticizing effects of water (*4*–*6*). Surface radiative cooling leads to a strong inversion layer close to the ground, limiting dispersion of pollutants largely from combustion sources (*7*). In Fairbanks, sulfate accounts for 15 to 25% of the wintertime PM_2.5_ mass concentration, making it the second most abundant species following organic aerosol (OA) compounds, which account for 40 to 70% and are largely from wood burning for domestic heating (*8*–*12*). Nitrate accounts for a small fraction (~5%) of PM_2.5_ mass concentration (*8*). Heating oil is a major source of sulfur at ground level (*8*). Primary sulfate accounts for about 62% of total sulfate (TS), with the remaining fraction formed through secondary chemical pathways (*13*).

Campbell *et al*. (*9*) showed that substantial amounts of secondary organo-sulfur species are formed in Fairbanks during the winter, with hydroxymethanesulfonate (HMS) being the most notable. HMS is a major component of S(IV), which includes HMS, HSO_3_^−^, SO_3_^2−^, and possibly other adducts. Total S(IV) concentrations can be present in large amounts during wintertime, between one quarter to one half of the sulfate molar concentrations during pollution periods. The chemistry of HMS was studied extensively in the 1980s (*14*, *15*). HMS is formed in atmospheric water through the uptake of HCHO and SO_2_ and the subsequent reaction of SO_3_^2−^(aq) with HCHO(aq) (*14*). HMS has been thought to form primarily in clouds or fogs, not in aerosols. The three to six orders of magnitude greater water content of a typical cloud droplet compared to aerosol-associated water provides highly increased medium for reaction that is effective in taking up reactive soluble gases and promotes higher pH values than for aerosols (*16*). Because of this, clouds and fogs are generally viewed as a more effective medium for secondary aqueous aerosol formation compared to fine PM (i.e., water associated with PM_2.5_ chemical components).

More recently, HMS has been observed in pollution events in Beijing during cold periods with high RH and extremely high PM_2.5_ mass concentrations or when clouds and fogs are in the region (*17*). Song *et al*. (*18*) suggested that HMS formation could take place in aerosol droplets in China due to high aerosol pH and ionic strength, and Wang *et al*. (*19*) estimate that about 36% of HMS measured in Beijing could be attributed to aerosol formation. Recent laboratory studies performed at room temperatures (~25°C) find that the HMS formation rate in aerosol water could be two to three orders of magnitude higher than in bulk water due to strong ionic strength effects, and HMS formation may be important if the pH is higher than 4 (*20*).

HMS formation is highly pH dependent because SO_3_^2−^(aq) is only present in sufficient amounts for notable HMS formation when pH is greater than ~4 (*9*, *14*). At 25°C, the lifetime against decomposition of HMS is a few hours at pH 6 and more than 100 days at pH < 3 (*15*, *16*, *21*). It can be converted to SO_4_^2−^(aq) by reaction with OH(aq), which reduces the lifetime of HMS and makes it a possible intermediary in secondary formation of SO_4_^2−^(aq) (*22*).

Like HMS chemistry, most aqueous sulfate chemical formation pathways are highly pH dependent (*20*, *23*–*25*). pH varies widely across the globe due to regional differences in key factors like temperature, RH, and local emissions that affect aerosol chemical composition. Fine-particle pH varies diurnally (*26*) and seasonally (*26*–*29*), inversely tracking temperature changes, with higher pH at night versus day and in the winter versus summer. Table S1 shows the extent of pH variation globally. Previous studies have used the partitioning of semi-volatile species (*30*–*32*) and temperature (*23*, *27*) to explain the variability of pH in different regions. Despite the key role of pH in aqueous phase chemistry, few studies on PM_2.5_ pH have been conducted for extreme cold conditions.

Here, we investigate the effect of low temperature on particle pH, which has a strong impact on secondary aerosol formation in supercooled aqueous particles. We examine the formation of unusually high concentrations of particulate HMS measured in Fairbanks during extreme cold because it only forms in liquid water through a highly pH-dependent process. The data are based on a large suite of measurements made during the 15 January to 28 February 2022 Alaska Layered Pollution and Chemical Analysis (ALPACA) campaign and analyzed using thermodynamic aerosol models ISORROPIA II and ISORROPIA-Lite (herein called ISOLITE), assuming particles in metastable state.

## RESULTS

### Fairbanks aerosol composition and ALWC

**Fig. 1.**
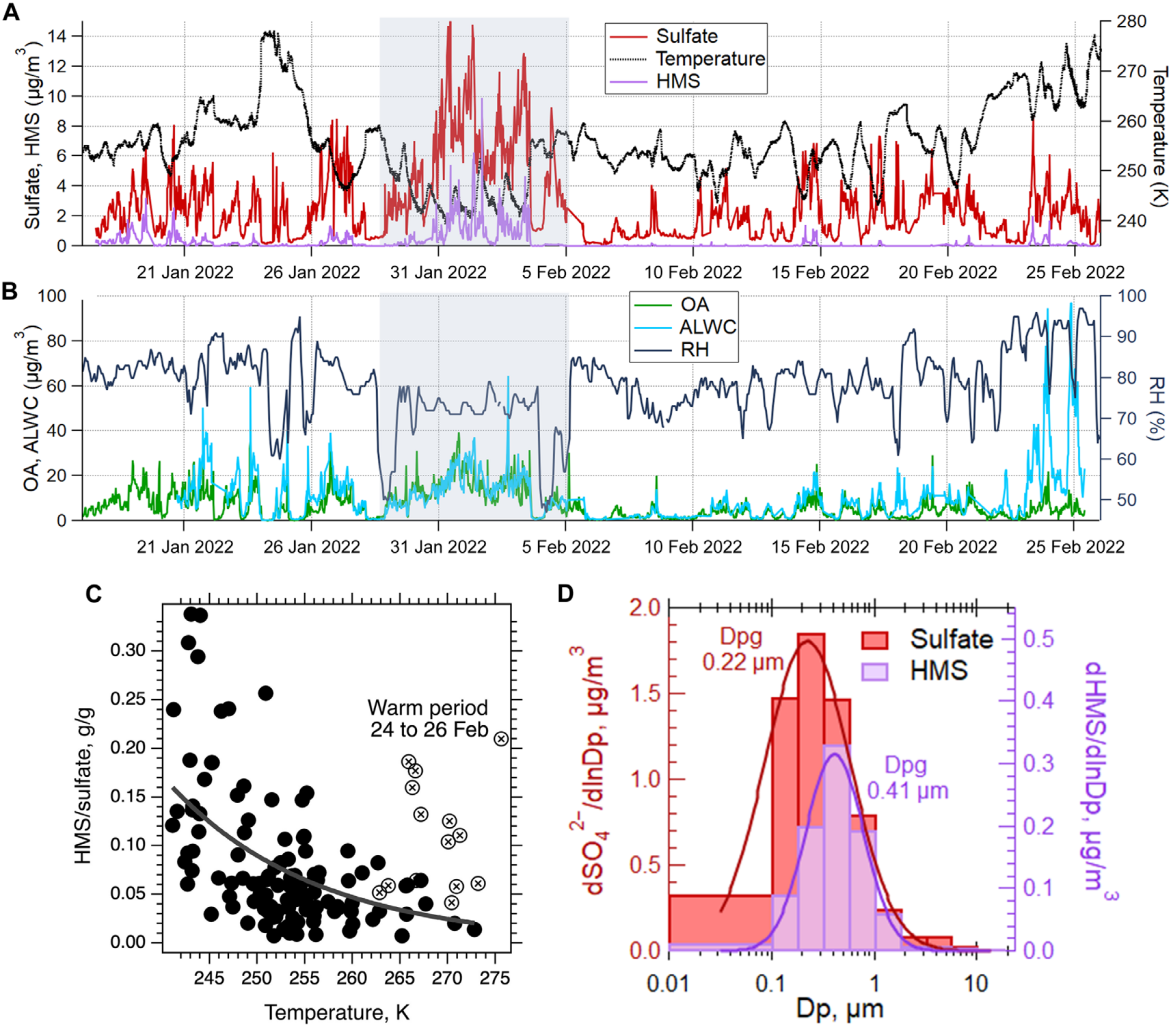
Variation of PM_2.5_ species and meteorological parameters during the ALPACA field study and characteristics of HMS relative to sulfate. Time series of (**A**) PM_2.5_ sulfate, PM_2.5_ HMS, and temperature; (**B**) PM_2.5_ organics, aerosol liquid water content (ALWC) calculated by ISORROPIA-Lite, and RH during the ALPACA campaign. The polluted period is highlighted in gray. HMS shown here is calculated as 70% of total S(IV) (see Materials and Methods). (**C**) Mass ratio of HMS to sulfate versus ambient temperature for PM_2.5_ and TSPs. Data denoted by points with X’s that do not follow the trend with temperature correspond to a warm period of high RH when cloud/fog influences may have occurred. (**D**) Size distributions of HMS and sulfate collected with a multistage cascade impactor (*54*, *62*) during the polluted period (10:00 a.m. 30 January to 9:00 a.m. 1 February), the lognormal fit with geometric mean particle diameter (Dpg; in micrometers) is shown for each).

During the ALPACA study, we recorded periods of high concentrations of all PM_2.5_ species, including sulfate and HMS during the coldest episodes. Figure 1 shows the time series of PM_2.5_ sulfate, HMS, OA, aerosol liquid water content (ALWC) computed by ISOLITE, RH, and ambient temperature during the ALPACA campaign period. The most abundant PM_2.5_ species by mass was OA with a mean concentration of 6.4 μg/m^3^, followed by sulfate at 2.7 μg/m^3^ (table S3). The non-refractory mass fractions [calculated using aerosol mass spectrometer (AMS) PM_1_ and aerosol chemical speciation monitor (ACSM) PM_2.5_] of OA (68 to 72%), sulfate (14 to 22%), nitrate (6 to 8%), and ammonium (3 to 7%) are similar to those reported in previous winters (*8*). Although fractional contribution of these components to fine PM mass is relatively invariant, there is large variability in the PM_2.5_ species concentrations, with high concentrations during the colder periods due to emissions from heating and strong temperature inversions near the surface. This is demonstrated by the polluted period from 29 January to 5 February shown in Fig. 1, which had the highest concentrations observed during the study and corresponded to a period of extreme cold and RH between 70 and 80%. HMS is notably highest during the event and is enhanced relative to other species; the HMS/sulfate molar ratio reached 0.37, a study maximum. There is a trend of a strong, nearly exponential increase in HMS relative to sulfate as temperature drops (Fig. 1C), with the exception of a period with unusually high RH (>90%, see Fig. 1B). ISOLITE predicts exceptionally high ALWC (up to 85 μg/m^3^, Fig. 1B and fig. S5) and higher aerosol pH, which may have contributed to high concentrations of HMS, reducing the effect of temperature in this case. We discuss aerosol pH further beginning in the next section. During the pollution event, HMS was also shifted to slightly higher sizes than sulfate (Fig. 1D). These observations can be explained by a large fraction of the sulfate being primary (*13*), with secondary HMS formation, promoted by lower temperature, in aqueous particles and leading to a shift to larger sizes.

Measurements of sulfate, ammonium, nitrate, and OA lead to a predicted study mean ALWC of 8.0 μg/m^3^. Attribution of the water to individual aerosol species (provided by ISOLITE) indicates that sulfates (all sulfate and bisulfate species) contribute to 47% of total ALWC, organic species 41%, and nitrates 12%. A recent study using a particle phase discriminator found that, during winter in Fairbanks, only 1.3% of near-surface cloud and fog particles (8 to 112 μm) contain liquid water, and only 0.3% contain liquid water during cold periods (below −30°C) (*33*). However, fine aerosols can remain as supercooled solution and contain liquid water (*3*, *4*). Because HMS can only be formed in liquid water, these data show a substantial enhancement in aqueous phase secondary aerosol production at lower temperature, occurring in supercooled water associated with PM_2.5_ at temperatures at least down to 238 K (−35°C).

### Aerosol pH and partitioning of TA and TN

**Fig. 2.**
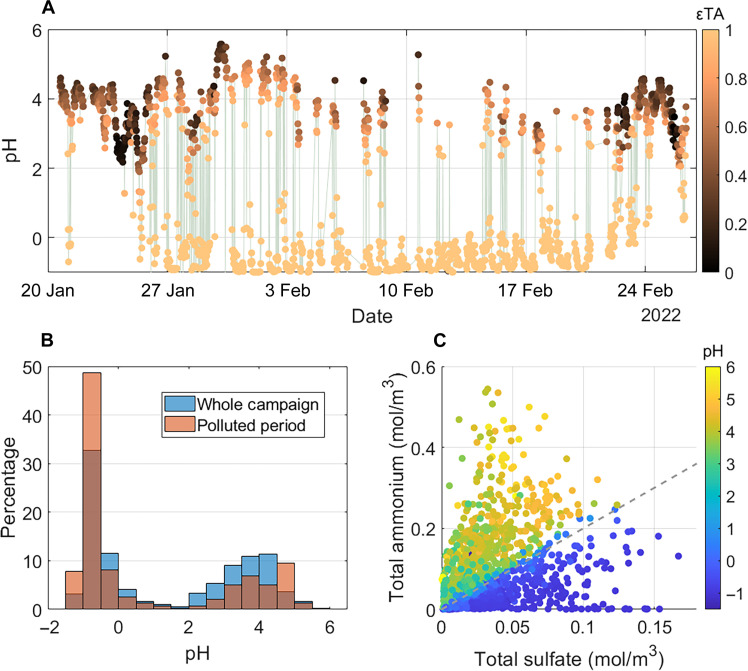
Summary of observations and behavior of fine-particle pH in Fairbanks and relationship to TA and sulfate. (**A**) Predicted pH time series during the campaign colored by molar ammonia partitioning (εTA), with the polluted period highlighted in gray with 30-min time resolution. (**B**) pH frequency distribution for the whole campaign and for the polluted period (29 January to 5 February). (**C**) Total ammonium (TA) versus total sulfate (TS) (i.e., HSO_4_^−^ + SO_4_^2−^), colored by pH, for the ALPACA campaign. Gray dashed line is 2:1 (a molar ratio of 2).

The pH of the liquid drops where HMS is formed plays a key role. Figure 2 shows fine-particle pH calculated by ISORROPIA II at 30-min time resolution using high-resolution measurements (see Materials and Methods). A unique behavior is observed: pH rapidly jumps between a high (3 to 5.5) and low (−1 to 1) range in a short period of time (Fig. 2A). Fine-particle pH in this study is largely controlled by the fraction of ammonium and sulfate. Nitrate can also affect particle pH, but, here, total nitrate (TN) molar concentrations are lower than sulfate by a factor of 2 (see section S4 for more information). We further examine the impact of HMS on aerosol acidity, as HMS is a nonvolatile strong acid (*34*). For this, we assume HMS as equivalent sulfate on a molar basis, which provides an upper limit on its effect on pH because HMS is less hygroscopic and can at most contribute one H^+^ per HMS, whereas sulfate can contribute up to two H^+^. As predicted by ISORROPIA II, the impact of HMS on aerosol pH is minimal, with 90% of the data having a pH reduced by 0.2 or less (fig. S6).

We also examine the effect of external mixing on calculated aerosol pH. Individual particle measurements showed that the number fraction of particles containing HMS increased with particle diameter (fig. S7), consistent with aqueous-phase formation (*35*, *36*). In addition to forming via secondary aerosol processes, sulfate and ammonium are also observed in primary combustion emissions (*37*–*39*), with the majority of the sulfate mass during ALPACA associated with primary combustion emissions (*13*). Individual particle analysis showed that 0.2 μm diameter was roughly the particle size for transition between smaller, newly emitted combustion particles that had not yet taken up water and larger particles that had taken up water and gained substantial secondary aerosol mass, including ammonium, sulfate, and HMS. This is consistent with the HMS geometric mean diameter by mass of 0.42 μm, compared to 0.21 μm for sulfate (Fig. 1C). The mainly non-deliquesced aerosols smaller than 0.2 μm account for the majority of the number concentration but only 40% of the PM_2.5_ sulfate mass and 25% of HMS mass. In contrast, the particles larger than 0.2 μm account for 60% of the PM_2.5_ sulfate and ammonium mass. The large extent of internal mixing between sulfate and ammonium, on a mass basis, is also demonstrated by the overlap of sulfate and ammonium mass size distributions from Micro-Orifice Uniform Deposit Impactor (MOUDI) measurements (fig. S8A) and the high correlations [correlation coefficient (*r*) > 0.95] between bulk PM_2.5_ sulfate and ammonium (fig. S8, B and C). We take this into account in the pH calculation by repeating the pH and liquid water calculations using only 60% of the PM_2.5_ sulfate and ammonium, the species that drive pH. Using either bulk PM_2.5_ or 60% produced similar predicted pH. Chloride and all nonvolatile cations (NVCs) except potassium were omitted from the pH calculations, as single-particle analysis showed that these chemical species were rarely in the same individual particles as the HMS (see section S2 and fig. S9 for calculations including all NVCs). Potassium was decreased to 75% based on MOUDI measurements from the size range 0.18 to 3.2 μm. All these analyses show a minor effect relative to using bulk PM_2.5_ concentrations on predicted pH, indicating that mixing state for this study has little influence on predicted pH of deliquesced aerosols (fig. S10).

One way to test the assumptions of mixing state, equilibrium, and the thermodynamic model predictions is to compare observed and calculated partitioning of a semi-volatile species. In this case, we focus only on NH_3_/NH_4_^+^, because nitric acid/nitrate were minor components of PM_2.5_ and measurement uncertainty due to low concentrations of gaseous nitric acid makes comparison difficult. εTA refers to the molar fraction of ammonium in the aerosol phase to the total of gas plus particle, NH_4_^+^/(NH_4_^+^+NH_3_). We find that the measured εTA is correlated with predicted values but lower by 50 to 60% (fig. S11, A and B), meaning that the model predicts more ammonium in the aerosol phase. The model can also predict the total ammonium (TA) to be entirely in the aerosol phase for cases where measurements indicate that it is mostly in the gas phase, and the measurements with the ACSM (PM_2.5_) agree better than the AMS (PM_1_). Some scatter may be due to varying effects of NVCs, other factors/species that affect pH not treated by the model, simplifications by the model, and measurement uncertainty. However, we believe that this is a reasonable agreement given the highly nonlinear behavior of εTA with pH, the populations of non-deliquesced aerosols (e.g., fresh soot) and those not represented by the composition investigated (e.g., dust), and uncertainty in the measurements (e.g., NH_4_^+^ measured by the ACSM and AMS; fig. S11, A and B). This agreement on partitioning further supports the metastable state of supercooled liquid particles.

On the basis of the thermodynamic analysis, Fig. 2A illustrates that ISORROPIA II predicts an εTA close to 0 when pH is above ~3, indicating that ammonia is mostly in the gas phase, whereas, at pH less than 3, εTA is close to 1 and all ammonia is in the particle phase. Thus, the largely bimodal pH distribution (Fig. 2B) is linked to the ammonium partitioning to the particle, where it is either mostly in the gas phase or mostly in the particle phase. The jump from low pH up to ~4 to 5, a more neutral fine-particle water (Fig. 2A), is important and unique among studied regions (table S1). The bimodal pH trend with more neutral pH upper condition can be explained by the drop in ammonia volatility with temperature and its impact on the buffering capacity of the multiphase pair NH_3_(g)/NH_4_^+^(aq) (also referred to as ammonia buffering).

Equilibrium partitioning of ammonia/ammonium to the gas/particle phase is highly dependent on temperature, as described by Guo *et al*. (*31*). See section S5 for details of the partitioning as it applies to the conditions of this study. The ammonia partitioning can be described by the following equation

$$\varepsilon \text{TA}=\frac{\left(NH_{4}^{+}\right)}{\left(NH_{4}^{+}\right)+\left(NH_{3}\right)}=\frac{\text{ALWC}\times R\times T\times K_{H}\times K_{a}\times \left[H^{+}\right]}{1+\text{ALWC}\times R\times T\times K_{H}\times K_{a}\times \left[H^{+}\right]}$$ (1)

(NH_4_^+^) and (NH_3_) are the concentration of aqueous particle ammonium and ammonia in air, respectively; ALWC is the aerosol liquid water content (e.g., gram of water per volume of air); *R* is the universal gas constant; *T* is the temperature; *K*_H_ is the Henry’s law constant; *K*_a_ is the acid dissociation constant of aqueous ammonia to ammonium; and [H^+^] is the liquid concentration of H^+^, which is related to pH. Both *K*_H_ and *K*_a_ substantially increase as temperature drops. For example, a change in temperature from 293 to 233 K (20° to −40°C) has a combined effect that increases the product of ALWC × *R* × *T* × *K*_H_ × *K*_a_ in Eq. 1 by four orders of magnitude (fig. S12C).

εTA when plotted against pH follows a sigmoidal-shaped curve (fig. S12D). The maximum buffering capacity is the condition where pH changes the least with the addition of an acid or base to the aqueous system (*32*); the maximum buffering capacity of TA occurs when εTA = 0.5. Figure S12D shows that, for a given amount of ALWC, if εTA is maintained at 0.5, then the equilibrium pH will change from about 1 to 5 as temperature drops from 293 to 233 K due to marked changes in *K*_H_ and *K*_a_ with temperature (fig. S11, A and B). In contrast to the buffering pair NH_3_(g)/NH_4_^+^(aq), for the other buffering pair sulfate/bisulfate [HSO_4_^−^(aq)/SO_4_^2−^(aq)], there is only a small temperature dependence on the acid dissociation constant, and, because it is nonvolatile, it is not affected by temperature-dependent Henry’s law partitioning. Figure 2C shows that this leads to a high sensitivity of aerosol pH to the TA-to-TS ratio (TA/TS) and higher pH levels in Fairbanks (fig. S12D) compared to many other regions with more moderate temperatures where pH is also mainly influenced by sulfate and ammonium.

**Fig. 3.**
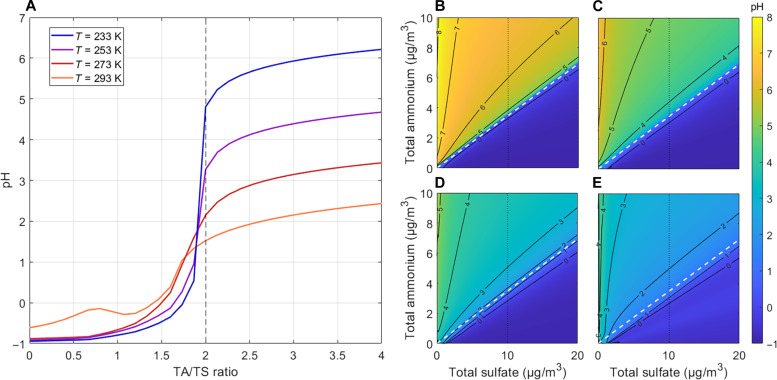
Sensitivity of fine-particle pH to TA and TS as a function of temperature. (**A**) The importance of how TA/TS changes with temperature. This can be visualized as the pH change on the dotted vertical line where TS = 10 μg/m^3^ in plots (B) to (E). (**B** to **E**) The pH dependence on TA and TS at varying temperatures, with the white dashed line indicating where the molar TA/TS = 2. (B) 233 K, (C) 253 K, (D) 273 K, and (E) 293 K. Plots (B) to (E) all have the same *x* axis and *y* axis, and all use the same pH colorbar shown on the right. Mean campaign masses of chloride, PM_2.5_ NVCs, and TN were used and held constant in all plots (tables S2 and S3). RH was held constant at 75%. The buffering pH range of sulfate decreases slightly as temperature decreases, while the ammonia buffering range increases markedly.

The ratio of TA/TS delineates when particle pH jumps between highly acidic to more neutral pH (Fig. 2C), which becomes more abrupt at colder conditions. Figure 3 shows pH as a function of TA and TS concentration at a range of temperatures. When the molar ratio of TA/TS < 2, ammonia is mainly in the aerosol phase (i.e., εTA is close to 1), and buffering from NH_3_(g)/NH_4_^+^(aq) is minimized. pH is then buffered by HSO_4_^−^(aq)/SO_4_^2−^(aq), and pH ranges from −1 to 1 (Fig. 3A). Hence, particle pH is controlled by the nonvolatile pair HSO_4_^−^(aq)/SO_4_^2−^(aq) when TA/TS < 2. In the case of Fairbanks, when TA/TS > 2, the pH is buffered by NH_3_(g)/NH_4_^+^(aq), resulting in a pH ranging from 3 to 5.5 (Fig. 3, A to C). Figure 3A also shows that regions with warmer temperatures (about 273 K and higher) do not have the same level of variation as colder regions like Fairbanks, where temperatures of 253 K and even down to 233 K are common in winter. This behavior has important ramifications. For example, in the summertime southeast US, which has an inorganic aerosol composition and ALWC similar to wintertime Fairbanks, PM_2.5_ has moved from a sulfate-dominated to ammonium-dominated regime over a period of years due to SO_2_ emission reductions. However, there is little change in aerosol pH when crossing the condition of TA/TS = 2 (e.g., crossing the white dashed line in Fig. 3E) due to much higher temperatures in the southeast US (*30*). In contrast, at very low temperatures (below 253 K; Fig. 3, A to C), a much larger and abrupt jump in pH occurs for the same change in TA/TS around TA/TS = 2, compared to higher temperatures. In all temperature scenarios, adding more nitrate or OA does not change this behavior (figs. S13 and S16), broadening the importance of this temperature effect on deliquesced aerosols to a wider range of conditions.

In addition to temperature, ALWC also affects the buffering of TA. An extreme case is when PM_2.5_ concentrations are exceedingly high. For example, over the North China Plain (NCP) with ambient temperature ~270 K and PM_2.5_ concentrations up to 400 μg/m^3^, ALWC can reach ~350 μg/m^3^ (*17*, *32*). At these unusually high ALWC, the buffering by TA versus sulfate produces pH values of 4 and 2, respectively, for an ideal solution (*32*). (For the sulfate buffering case, the pH is likely closer to −1 when non-ideality is considered.) This pH range is comparable to what we observe at very low temperature; thus, very high ALWC can produce the same effect as very low temperature on ammonia buffering. Note that a lower pH was not observed over the NCP in the study of Zheng *et al*. (*32*) because their high predicted concentrations of ammonia placed it in an ammonia-dominated regime for the whole study period (*24*, *32*). Thus, what we observe in wintertime Fairbanks is distinct from other studies. Conditions for rapid pH change are not possible at more moderate temperatures due to the thermodynamic behavior of the TA/TS system, except for regions with very high ALWC.

**Fig. 4.**
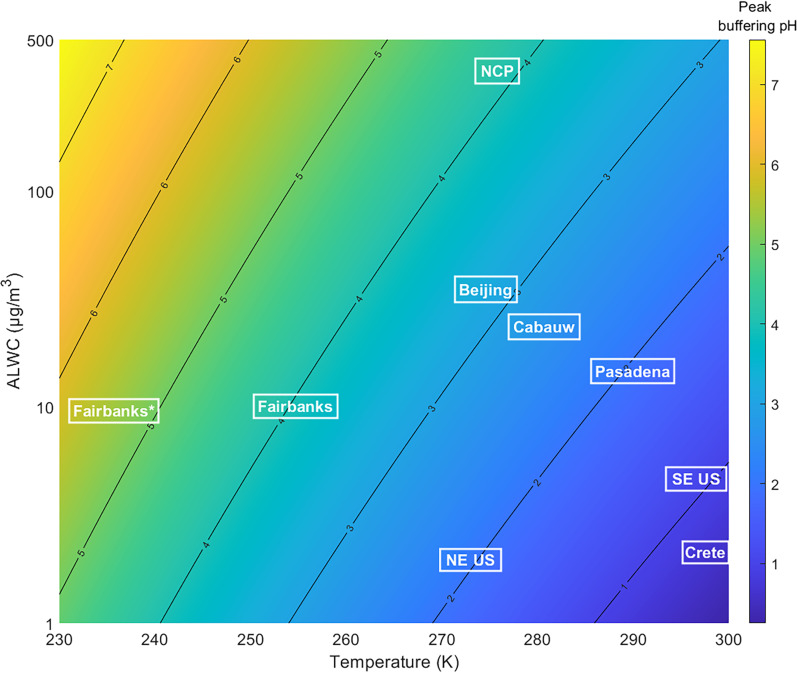
Fine-particle acidity at which ammonia gas-particle partitioning provides maximum buffering as a function of aerosol water and ambient temperature. pH associated with peak ammonia buffering (εTA = 0.5) versus temperature and ALWC, plotted with mean values for various campaigns that calculated ALWC. Fairbanks, Alaska, is this campaign, with no organic water included in ALWC. Fairbanks* is this campaign at the lowest measured temperature and no organic water. NCP are conditions for severe winter haze in China (*24*, *32*). Beijing, China, is typical wintertime conditions (*63*). Cabauw, The Netherlands, is the yearly mean conditions (*29*). Pasadena, California, is mean summertime conditions (*31*). Southeast (SE) US is mean summertime conditions in Centreville, Alabama (*26*, *30*). Crete, Greece, is for fall conditions (*64*). Northeastern (NE) US is wintertime aircraft measurements (*43*).

A broad comparison showing the sensitivity of pH at peak ammonia buffering (i.e., pH when εTA = 0.5) to ALWC and temperature, resulting from Eq. 1, is shown in Fig. 4. The contrast noted above between Fairbanks and the NCP can be seen on this plot. As another example, consider two studies in locations that have similar temperatures, the northeast US and Beijing, but with the largest difference in ALWC, 2 to 35 μg/m^3^, respectively (not including the NCP). The roughly order of magnitude greater ALWC is associated with a change in pH at peak ammonium buffering of about 1.1 pH units. It takes another order of magnitude increase in ALWC to increase pH one more unit from that seen in Beijing to that of the NCP (35 to 350 μg/m^3^). In contrast, two sites that have similar ALWC, Fairbanks and Pasadena, have temperatures of ~255 K versus 290 K, respectively, and are associated with a pH change of ~2 units, about twice as much as that caused by a one order of magnitude difference in ALWC. The coldest temperature during the Fairbanks study was the polluted period (237 K, see Fig. 1), which is associated with pH at TA buffering of ~5.5 compared to the study average pH of 4. Although not all locations shown in Fig. 4 are ammonia sulfate–dominated, it demonstrates how pH associated with ammonia buffering changes from region to region and that there is a much higher sensitivity to changes in temperature than in ALWC.

## DISCUSSION

We have shown that, for fine aerosol particles in equilibrium with semi-volatile species such as ammonium, temperature can largely facilitate certain types of aerosol chemistry by inducing marked changes in aerosol acidity. The detection of HMS in the past three Fairbanks winters and the pH predictions carried out here and in past studies (*9*) corroborates this effect, even at temperature down to −35°C when particles are still in metastable state. Other factors, of course, contribute to high HMS concentrations in Fairbanks winter. Low oxidant concentrations would reduce HMS loss by reaction with OH and maximize HMS production rate by limiting loss of HSO_3_^−^/SO_3_^2−^ by reaction with other oxidants (O_3_, H_2_O_2_, and NO_2_). High ionic strengths in the concentrated aqueous solutions associated with PM_2.5_ water could also enhance HMS formation rates (*20*). Together, these factors promote HMS formation in Fairbanks, but the parameter that truly enables the substantial HMS production in PM_2.5_ is low temperature and its effect on pH and heterogeneous chemistry. This simple but clear link carries important and broader implications.

There are potentially specific effects on Fairbanks air quality. At extremely low temperature, aerosol pH can be very sensitive to TA/TS, and this results in a self-limiting production of certain secondary aerosol species, such as HMS. Starting with a relatively high amount of TA relative to sulfate (TA/TS > 2), pH tends to be high (especially at extremely low temperatures), and the formation pathways for sulfate and HMS involving SO_3_^2−^ are facilitated. Upon formation of sulfate and HMS, TA/TS decreases and pH rapidly becomes highly acidic (pH ranging between −1 and 1). Once TA/TS < 2, the equilibrium shifts away from SO_3_^2−^ in the supercooled aerosol, shutting off these particle formation routes. The pH may then increase again due to local emissions of NH_3_(g). The unique behavior of aerosol pH may also have important implications for Fairbanks sulfur emission control strategies. As of 1 September 2022, all fuel oil sold in Fairbanks was required to contain less than 1000–part-per-million (ppm) sulfur, such that #2 heating oil could no longer be used for home heating. The new regulation may substantially reduce gaseous SO_2_ and primary sulfate emissions but with no change in TA. This means that longer periods of moderate aerosol pH (4 to 6) would arise, facilitating the formation of S(IV) (e.g., HMS) and S(VI) (e.g., sulfate), partially offsetting gains in sulfur emission reductions.

These findings also have implications beyond Fairbanks, given that the major fine-particle–neutralizing cation worldwide is semi-volatile ammonium. It is recognized that large amounts of ALWC can also raise aerosol pH relative to that of fine particles and increase secondary aerosol formation, such as in clouds and fogs or in highly polluted regions with high RH. Measurements of HMS formation over the NCP demonstrate this. However, regions of very high ALWC may not be as globally widespread as those of low temperature and moderate and higher RH, where particles could be in a supercooled liquid state. Such regions that also have emissions leading to secondary aerosol formation could include urban or industrialized regions located at higher latitudes during cold seasons or when plumes near the surface are lifted to high altitudes. Deep convection of fire emissions in regions of intensive biomass burning is an example of the latter and is increasingly prevalent. Furthermore, just as oxalate is often interpreted as a tracer for cloud processing (*40*, *41*), the presence of HMS indicates a very specific range of conditions during the air parcels history, making it a potentially valuable marker of such conditions for secondary processing. The temperature effect on particle acidity that we show here occurs over a continuum of temperature and is most obvious in extreme cold; the substantial HMS formation observed in Fairbanks winter during extreme cold periods provides clear evidence of it. Unlike ALWC-driven pH buffering, ammonia buffering at low temperatures becomes highly sensitive to the total amount of gas-particle precursors (i.e., the buffering capacity becomes increasingly less effective as temperature decreases), which affects the overall extent of secondary aerosol formation through pH-dependent reactions in fine-particle water. We therefore have identified an important effect on fine-particle pH that has widespread implications. While at warmer temperatures, semi-volatile ammonia (and its buffering effect) stabilizes aerosol pH, which renders it insensitive to TA/TS (*20*, *27*, *29*), at low enough temperatures, the buffering effect goes away owing to its limited volatility, and pH becomes much more sensitive to TA/TS. This effect of temperature applies of course to other semi-volatile species (e.g., inorganic nitrate and chloride) as well.

## MATERIALS AND METHODS

### Measurements

All measurements were conducted in trailers near the UAF CTC building in downtown Fairbanks (University of Alaska Fairbanks Community and Technical College, 64.84064°N, 147.72677°W, elevation 136 m above sea level) from 17 January to 25 February 2022, unless stated otherwise. More information regarding details of the ALPACA campaign can be found in (*42*). Figure S1 shows the CTC site in relation to the other sites that are mentioned below.

High time-resolution online measurements were done for various anions and cations. Particle-phase sulfate, S(IV), nitrate, and chloride were measured using a particle into liquid sampler coupled with ion chromatography (PILS-IC) at 23-min time resolution. PILS-IC has been deployed in many other particle pH studies, including WINTER, CalNex, and SOAS (*26*, *31*, *43*). PILS-IC also measured phosphate, acetate, formate, and oxalate, but measurements for these species were mostly below detection limit of roughly 0.05 μg/m^3^. The PILS sample tubing was stainless steel and was located about 4 m above the snow-covered ground. It was fitted with a PM_2.5_ cyclone, had a nominal flow of 16.7 liters/min, and was denuded of gases by a series of activated carbon denuder and sodium carbonate–coated glass honeycomb denuder. More details regarding the PILS setup can be found in (*9*).

TN and total chloride were measured using a mist chamber sampling at nominally 25 liters/min coupled to an anion IC (MC-IC) (*44*, *45*). TA was measured with the same MC coupled to a cation IC. Both the anion and cation systems ran at ~25-min time resolution. They shared the same inlet, which was about 4 m above the snow-covered ground. The inlet was a short (~60 cm long) straight 3/8-inch (9.525-mm) OD Teflon tube inside a stainless-steel tube that was heated to about 10°C above ambient to limit ammonia gas wall losses. The anion MC-IC was not run during the last week of the campaign, so PILS-IC nitrate was used in the ISORROPIA II TN input for this period. The mean nitrate measurements for MC-IC and PILS-IC are within 5% of each other during the period when they were both running, so we expect gas-phase HNO_3_ concentrations to be negligible.

The distribution of sulfate, ammonium, oxalate, and HMS across the submicron aerosol population was examined through single-particle measurements using an aerosol time-of-flight mass spectrometer (ATOFMS) in a residential neighborhood located ~2.4 km east-northeast of the CTC site (house site, fig. S1). The ATOFMS, described in detail by Pratt *et al*. (*46*) and Gunsch *et al*. (*47*), measured the chemical composition and size of individual aerosol particles from 0.1 to 1.0 μm *d*_va_ (vacuum aerodynamic diameter). For the analysis herein, size-resolved number fractions were calculated for individual particles containing sulfate [mass/charge ratio (*m/z*) −97 (HSO_4_^−^)] (*48*, *49*), ammonium [*m/z* 18 (NH_4_^+^)] (*50*), oxalate [*m*/*z* −89 (C_2_HO_4_^−^)] (*51*), and/or HMS [*m/z* −111 (HOCH_2_SO_3_^−^)] (*52*, *53*). Although we expect that HMS is made possible by aqueous ammonium raising the aerosol pH, single-particle analysis showed that some HMS-containing particles did not contain ammonium. HMS-containing particles lacking ammonium may be an artifact caused by loss of semi-volatile species (e.g., water and NH_4_^+^), during indoor measurements of air sampled from an extreme cold environment. An increase in sample temperature mainly affects smaller particles due to the Kelvin effect, whereas there would be no effect on nonvolatile sulfate and HMS.

Two sets of filter data were examined. The first set of filters measured total soluble particles (TSPs) and were run for 12 hours during cleaner periods and about 6 hours during polluted periods. The second set measured PM_2.5_ and collected particles for ~24 hours. Section S2, fig. S2, and table S2 compare the various NVCs for PM_2.5_ and for TSP.

Nominally 2-day MOUDI samples were collected and analyzed for the suite of anions and cations. MOUDI data showed that a majority of the NVCs in PM_2.5_ were in size ranges greater than 1 μm particle aerodynamic diameter.

Both the TSP and PM_2.5_ filters used a hydrogen peroxide (H_2_O_2_) treatment to quantify HMS from total S(IV) (*54*). This method was used previously to convert free S(IV) species like sulfite and bisulfite to sulfate while leaving HMS intact (*55*). HMS is then quantified as the S(IV) after the H_2_O_2_ treatment. For the TSP filters, the treatment was done by manually adding 10 μl of 3% H_2_O_2_ to a 5-ml aliquot of the filter extract. This solution was shaken briefly and allowed to sit for 10 min before IC analysis. For the PM_2.5_ filters, the treatment was set up as a continuous flow injection system that mixed flow at a ratio of five parts extract to one part 6-ppm hydrogen peroxide solution. This flow passed through super-serpentine reactors with a residence time of ~7 min before entering IC for analysis. In both the TSP and PM_2.5_ filters, HMS was found to vary between 49 and 70% of total S(IV), depending on the range of particle sizes included. For PM_2.5_, the ratio was 70%, which is used to estimate HMS from online PILS-IC measurements here.

PILS, MOUDI, and PM_2.5_ filters all used the same IC method and Metrohm 761 IC units (Metrohm USA, Riverside, FL). Anions were measured via conductivity detection with a Metrosep A Supp-5 150/4.0 anion column (0.7 ml/min, 10.5 MPa) with a 3.2 mM sodium carbonate and 1 mM sodium bicarbonate eluent at pH of 10.5. A sample of 250 μl (+750-μl wash) was injected onto the column from a sample loop. Minor overlap in the S(IV) and S(VI) peaks is estimated to result in less than 20% error in S(IV) concentrations. More details are given in (*9*). For TSP, filters were analyzed immediately at the University of Alaska at Fairbanks, near the sampling site. Anions were analyzed using a Dionex AS-11 column with an eluent of 10 mM NaOH at a flow rate of 1.0 ml/min. An Anion Self-Regenerating Suppressor (ASRS) was used in the recycle configuration. Sample was loaded onto the column from a 250-μl sample loop. This IC system separated the S(IV) and S(VI) peaks with no overlap.

A high-resolution time-of-flight AMS (HR-ToF-AMS; Aerodyne Research Inc., USA; herein referred to as AMS) equipped with a PM_1_ aerodynamic lens and the original standard vaporizer was run in a trailer adjacent to PILS-IC and MC-IC. The sample RH measured at the AMS inlet was below 20% over the whole measurement period. Standard calibration procedures using 300-nm size-selected dried ammonium nitrate and ammonium sulfate were carried out at the beginning and the end of the campaign to determine the ionization efficiency of nitrate and relative ionization efficiencies of ammonium and sulfate. The composition-dependent collection efficiency, described by Middlebrook *et al*. (*56*), was applied to account for the bouncing effect on the vaporizer. Scatterplot of the total AMS mass concentrations versus mass concentration calculated from colocated scanning mobility particle sizer and PM_1_ black carbon measurements gave a good agreement [slope of 1.06; *R*^2^ (coefficient of determination) of 0.9].

An ACSM (Aerodyne Research Inc., USA) was run in a trailer next to the Alaska Department of Environmental Conservation’s NCore site, about 300 m away from CTC (fig. S1). The ACSM was equipped with a PM_2.5_ aerodynamic lens and capture vaporizer (*57*–*59*). A PM_2.5_ cut cyclone (URG) at ambient temperature was attached to the inlet, and the sample RH was maintained below 30% using a Nafion dryer located inside the sampling building just upstream of the ACSM.

Temperature measurements were made every minute. We use RH data recorded by the Alaska Department of Transportation (ADOT; https://erddap.aoos.org/erddap/tabledap/index.html?page=1&itemsPerPage=1000). Data were collected every 1 to 2 hours at a site near downtown Fairbanks (147.7104°W, 64.8353°N), about 600 to 700 m away from the CTC site (fig. S1). Figure S3A shows a reasonable agreement between the RH measured at the ADOT site and the ALPACA farm site (*42*). Although there is some variation between the two sites, most notable when the farm site has periods of much higher pH compared to the ADOT site, fig. S3B shows that this does not lead to an appreciable difference in the calculated pH. ADOT RH measurements are used because of its closer proximity to the CTC site and wider date range. Three points that were missing from the ADOT time series were filled in by the farm site RH.

Unless noted otherwise in the text, PM_2.5_ sulfate and nitrate measurements were made with a PILS, total of ammonia and ammonium (TA) with a mist chamber, and OA mass concentrations with an ACSM.

### Modeling

ISORROPIA II version 2.3 (http://isorropia.epfl.ch) is a thermodynamic equilibrium model used to calculate ALWC and pH in bulk PM_2.5_ (*60*). It was used for the majority of the calculations shown in the results section. Additional pH calculations were done using ISORROPIA-Lite (ISOLITE; http://isorropia.epfl.ch) to account for ALWC contributions from OAs. ISOLITE is nearly identical to the solution process of ISORROPIA II version 2.3 but uses precalculated tables for the binary activity coefficients, assumes metastable aerosol, and allows the water uptake from internally mixed hygroscopic organics to affect the semi-volatile partitioning of inorganic species considered by ISORROPIA II. The water uptake of the organics is parameterized using the hygroscopicity parameter (κ) and κ-Kohler theory and is described in detail by Kakavas *et al*. (*61*).

In this study, pH is defined as

$$\text{pH}=-{\text{log}}_{10}\;\left(\gamma m_{H+}\right)$$ (2)

where γ is the hydrogen ion activity coefficient and *m*_H+_ is the molality of the free H^+^ in solution (*23*). ISORROPIA II and ISOLITE assume that γ = 1 for simplicity. The ISOLITE results are noted explicitly.

ISORROPIA II and ISOLITE were run in forward mode. They both used the inputs of PILS sulfate, MC TN, MC TA, temperature, and RH. Additional calculations were done using ISORROPIA II with and without NVCs from PM_2.5_ filters. ACSM PM_2.5_ OA was the primary input for ISOLITE organics because it was higher than AMS PM_1_ OA and would provide an upper limit for ALWC estimates. Missing values in the ACSM data were filled in by AMS data when possible. The addition of organic species to overall ALWC for pH calculations do not produce any appreciable difference in pH compared to the calculations without organic ALWC (section S3 and fig. S4).

## Supplementary Materials

### This PDF file includes:

Sections S1 to S5 Figs. S1 to S17 Tables S1 to S3 References
