# Supplementary material for: Enhanced aqueous formation and neutralization of fine atmospheric particles driven by extreme cold
Source: Sci Adv. 2024 Sep 4;10(36):eado4373. doi: 10.1126/sciadv.ado4373 (PMC11421654; doi:10.1126/sciadv.ado4373)
Supplement: Supplementary file 1 — Sections S1 to S5 Figs. S1 to S17 Tables S1 to S3 References [file sciadv.ado4373_sm.pdf]

Science Advances
AAAS

Supplementary Materials for

Enhanced aqueous formation and neutralization of fine atmospheric particles driven by extreme cold

James R. Campbell *et al.*

Corresponding author: Jingqiu Mao, jmao2@alaska.edu; Rodney J. Weber, rweber@eas.gatech.edu

Sci. Adv. **10**, eado4373 (2024)
DOI: 10.1126/sciadv.ado4373

This PDF file includes:

Section S1 to S5
Figs. S1 to S17
Tables S1 to S3
References

Supplementary Text

S1. Instrument Comparison.

Table S3 shows measurements from different instruments. PILS sulfate and nitrate mean values agree with NCore filter mean values. Although ACSM and AMS agree with each other relatively well, they are both lower than PILS and NCore measurements, indicating possible sampling loss. Other reasons for these differences could be due to the lower size cut of the AMS, the presence of refractory sulfate and nitrate, and because the ACSM was not colocated with the PILS and AMS. Total ammonium measured by MC-IC is much higher than AMS, ACSM, and NCore ammonium measurements, which suggest a large portion of total ammonium is in the gas phase.

S2. Effect of Non-Volatile Cations.

Ca^{2+} and Na^+ were mainly in the coarse mode ($PM_{2.5\text{-}TSP}$). Ca^{2+} decreased from 0.23 μg/m^3 in TSP, where it was the most prevalent species by mass, to 0.025 μg/m^3 in $PM_{2.5}$, nearly 10x less. Sodium also decreased, from 0.20 μg/m^3 in TSP to 0.060 μg/m^3 in $PM_{2.5}$, almost 3.5x less. K^+ only decreased by about a third from TSP to $PM_{2.5}$ since it can have sources associated with mineral dust in the coarse mode and biomass burning (residential heating with wood) in the fine mode. Because of this, K^+ went from being the third largest NVC in TSP (25% by mass), to being the largest NVC species in $PM_{2.5}$ (48% by mass). Mg^{2+} has similar low concentrations in both size ranges. Overall, NVCs are about three times less in $PM_{2.5}$ than in TSP.

NVCs contributed less than one tenth of all cations by mass (on average 0.005 μmol/m^3 in $PM_{2.5}$), and had little effect on the pH of aqueous $PM_{2.5}$ particles. We compare bulk pH calculations using all NVCs (pH_{allNVC}) with bulk pH calculations using no NVCs (pH_{noNVC}) in Figure S9. Although there is some variability, 89% of the two datasets are within 1 unit of pH of each other. The addition of NVCs can help increase the pH to the higher pH range described in the main text, but it does not raise the pH higher than the buffering capacity of $NH_3(g)/NH_4^+(aq)$. Note that we only use potassium for the pH results described in the main text.

S3. Effect of Organics.

In Fairbanks, where organic $PM_{2.5}$ is the major pollutant, organic aerosols (OA) can contribute up to an estimated 50-60% to ALWC, and in this study contributed about 30% (not accounting for organosulfur compounds like HMS). Zheng et al. (*29*) found that ALWC was the biggest reason for pH differences in the SE US and the NCP. Analysis on organic ALWC was done by making a contour plots at OA = 60 μg/m^3 (Figure S17). Even at such high levels of OA (the mean OA mass concentration during the ALPACA campaign was 6 μg/m^3), which contribute up to 19 μg/m^3 ALWC, the buffering ranges of ammonia and sulfate are only slightly increased. The determining factor for pH in these cases are still the TA/TS ratio.

S4. Effect of Nitrate.

In Figure 3, we used a total nitrate concentration equal to the mean over the campaign, which was 1.0 μg/m^3. pH and ALWC contributions from nitrate were found to be insignificant for the conditions during the ALPACA campaign, due to low total nitrate (TN) concentrations relative to TS, and a peak buffering capacity around pH -3, which was overshadowed by the self-buffering of water at extremely low pH. Figure S13 shows how the peak buffering capacity of $HNO_3(g)/NO_3^-$

(aq) varies with temperature and ALWC. Figure S14 shows nitrate levels at a) 10 μg/m^3 (approximately half of the highest sulfate measurement) and b) 20 μg/m^3 (approximately equal to the highest sulfate measurement). In Figures 3 and S13 the same overall trends are observed regardless of nitrate concentration. However, as nitrate concentration increases, the amount of ammonia needed to neutralize the aerosol increases. Since the buffering pH of nitrate is around -3 to -2 in the temperature conditions observed in Fairbanks and nitrate concentrations are low, the self-buffering of water prevents nitrate decreasing the aerosol pH below -1. At higher temperatures observed during this campaign, the buffering range is around -1 to 0, which is similar to sulfate's, so the low pH range stays consistent. This low buffering range is consistent with the WINTER campaign, which was nitrate dominated and saw temperatures as low as -21 °C (*20, 39*).

The pH of Fairbanks $PM_{2.5}$ is mainly affected by ammonium and sulfate, with low levels of nitrate. This is not the case in all pH studies, where nitrate can be at similar or much higher levels than sulfate. However, we find that at low temperatures, nitrate will act in a similar role to sulfate due to the decreased volatility. Figure S15a shows that at low temperatures (233 K and 253 K especially), higher concentrations of nitrate will simply shift the inflection point of TA/TS ratio higher, while Figure S15b shows that if the TA/(TS + 1/2*TN) ratio is used at lower temperatures the inflection point remains at 2. However, as temperature increases and nitrate volatility increases, more nitric acid shifts to the gas phase at low pH, which reduces the importance of nitrate on aerosol pH and the TA/TS ratio. This is especially clear in Figure S15 at 293 K.

The results are similar in regions where sulfate is at very low concentrations. Figure S16 shows pH vs TA/TN ratio at different temperatures when TS is held constant at 1 μg/m^3. At 233 K, the pH changes sharply when TA/TN = 1, and the pH range is similar to an ammonium-sulfate dominated system. However, in contrast to sulfate, the volatility of nitrate increases with temperature. This causes the pH buffering range of nitrate to increase with temperature. This is especially obvious in the plots for 273 K and 293 K, where the "low" pH range of nitrate is similar to the "high" pH range of ammonium. At higher temperatures, nitrate volatility becomes a larger factor, but at regions where the temperature is much colder like Fairbanks it behaves in a similar manner to sulfate.

<u>Section S5. Partitioning of Ammonia/Ammonium.</u>

In the following equations, brackets [ ] represent the concentration of a species in the water, e.g., mole/mL, parentheses ( ) represent the concentration in air in, e.g. mol/cm^3 air, and we assume the density of water is 1 g/mL:

$$NH_3(g) \rightleftharpoons NH_3(aq) \qquad \text{(R S1)}$$

$$K_H = \frac{[NH_3]}{p_{NH3}} = \frac{[NH_3]}{(NH_3)\times RT} \qquad \text{(Eq S1)}$$

$$NH_3(aq) + H^+ \rightleftharpoons NH_4^+(aq) \qquad \text{(R S2)}$$

$$K_a = \frac{[NH_4^+]}{[NH_3][H^+]} \qquad \text{(Eq S2)}$$

K_H is the Henry's law constant, p_{NH3} is the partial pressure of $NH_3(g)$, R is the Universal Gas Constant, T is the temperature, and K_a is the $NH_{3(aq)}$ dissociation constant. When combined, these

equations give the molar ratio of particle phase ammonium to gas phase ammonia concentration, both in units of mole per volume of air, and εTA, assuming the density of water is 1 g/mL:

$$\frac{(NH_4^+)}{(NH_3)} = ALWC \times RT \times K_H \times K_a \times \left[H^+\right] = ALWC \times RT \times K_H \times K_a \times 10^{-pH} \qquad \text{(Eq S3)}$$

The Henry's law constant of ammonia increases from 60 M/atm at 298 K to $3.1*10^3$ M/atm at 233 K, by two orders of magnitude (*65*). Similarly, the acid dissociation constant of NH_3(aq) + H^+ ⇌ NH_4^+(aq) increases from $1.8*10^9$ M^{-1} at 298 K to $6.4*10^{11}$ M^{-1} at 233 K, by another two orders of magnitude (*66*). As a result, the equilibrium pH of ammonia increases by 4 units for a temperature change from 293 K to 233 K when εTA is 0.5 (Figure S12).

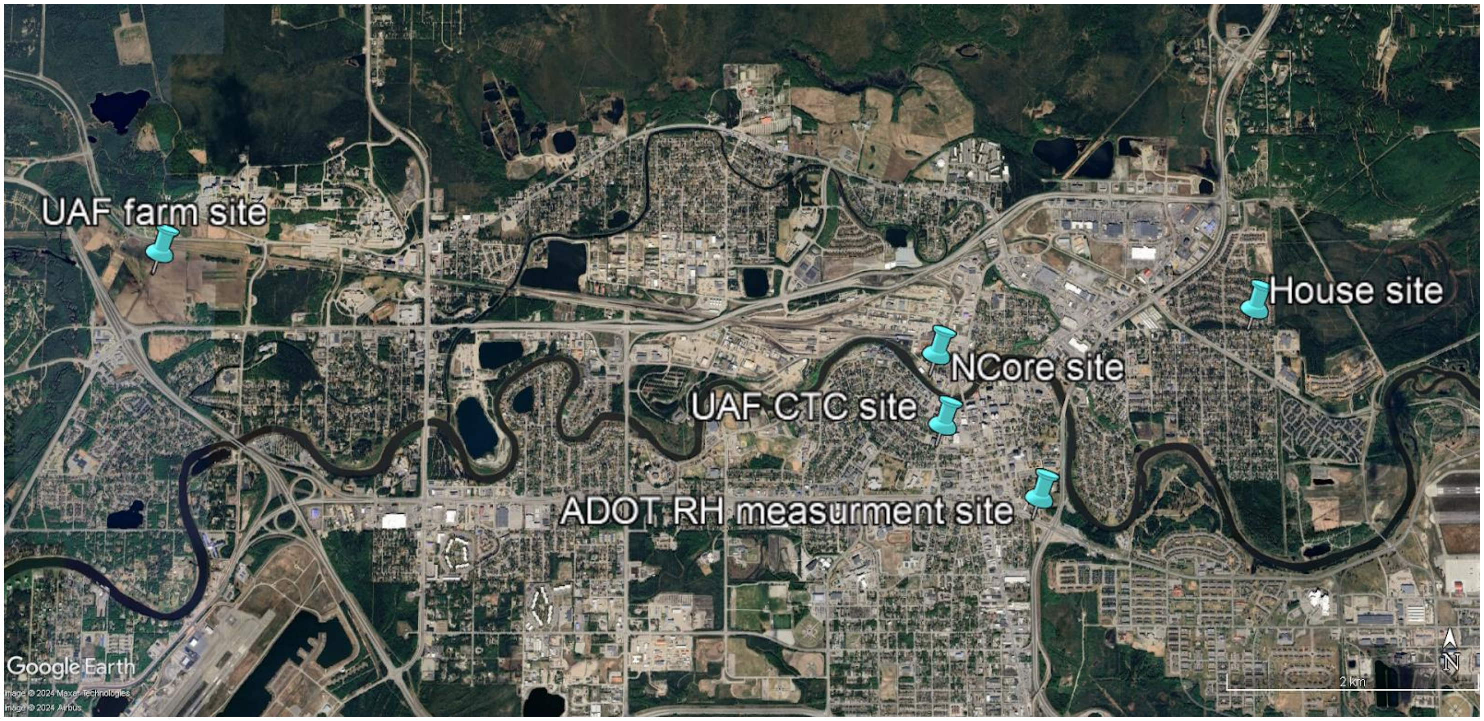

Figure S1. Map of the measurement sites.

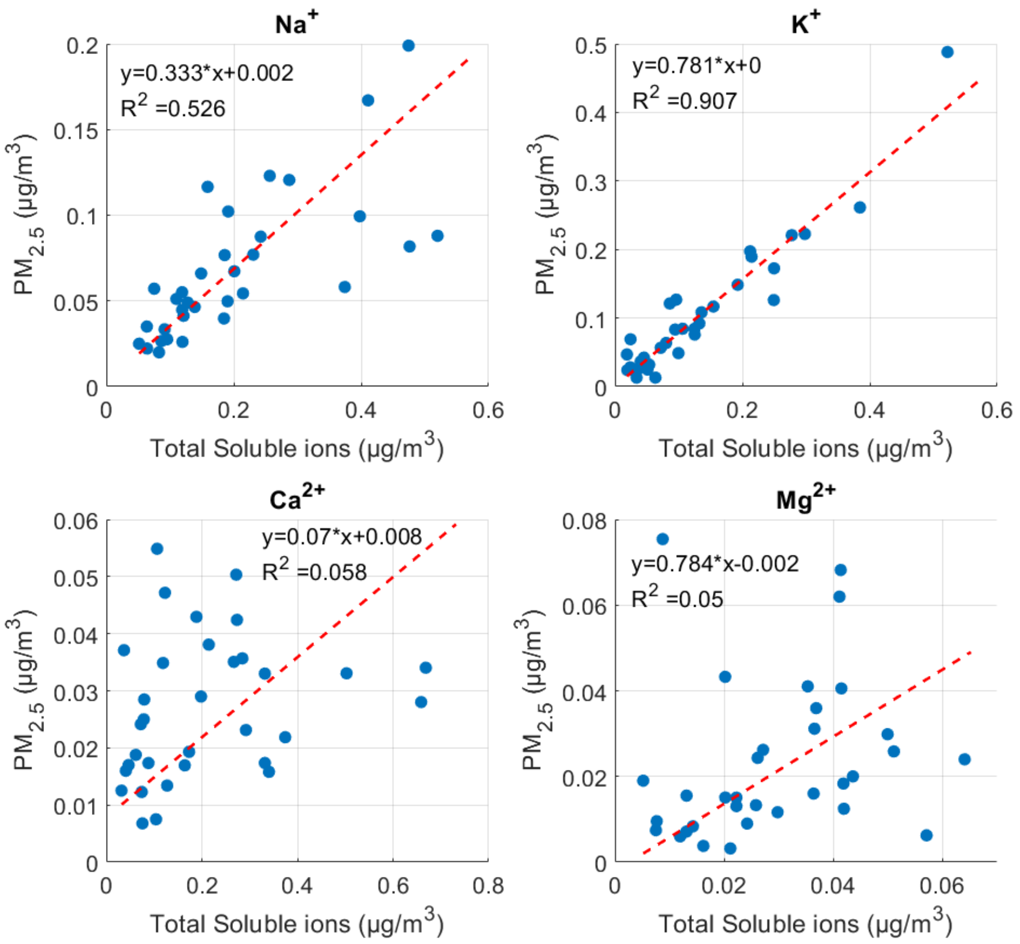

Figure S2. Comparison of $PM_{2.5}$ nonvolatile cations (NVCs) with total soluble particle (TSP) NVCs. York fit is applied here.

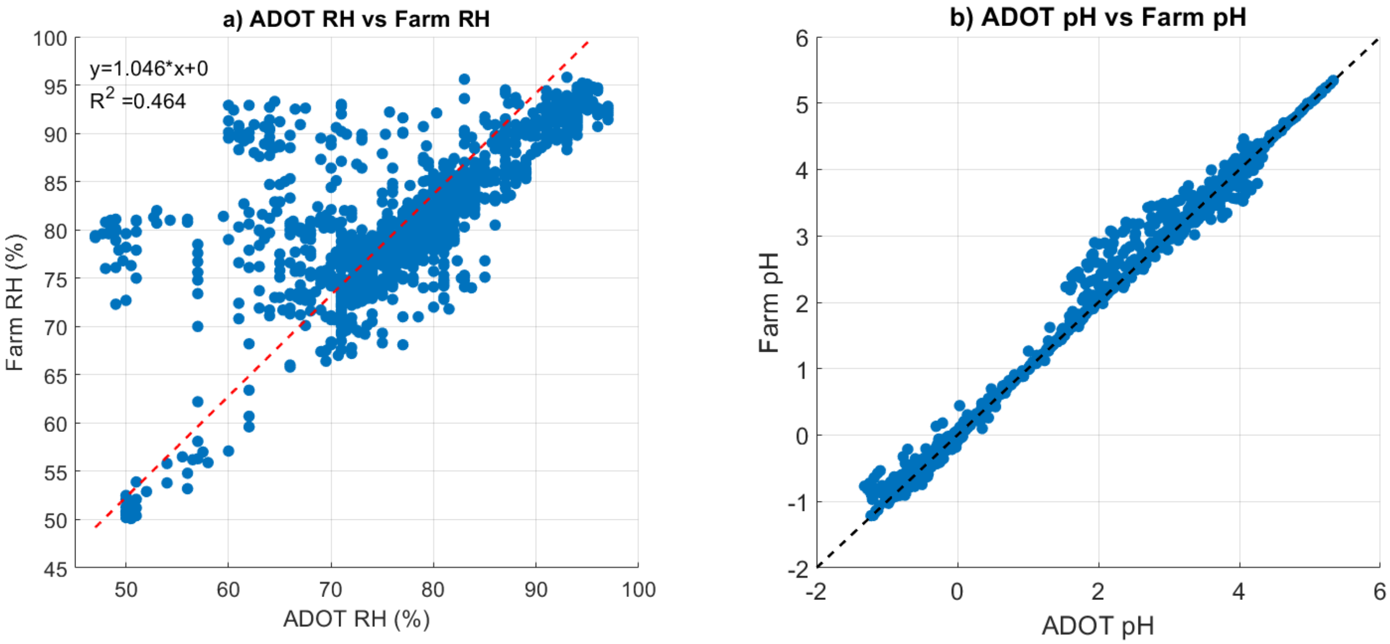

Figure S3. Comparison of relative humidity (RH) between the farm site and Alaska Department of Transportation (ADOT) site, and its effect on pH. a) Comparison of the relative humidity at the farm site vs ADOT site. York fit applied here with regression forced through zero. b) Comparison of pH calculated using farm site RH vs ADOT RH. Black line is 1:1 line.

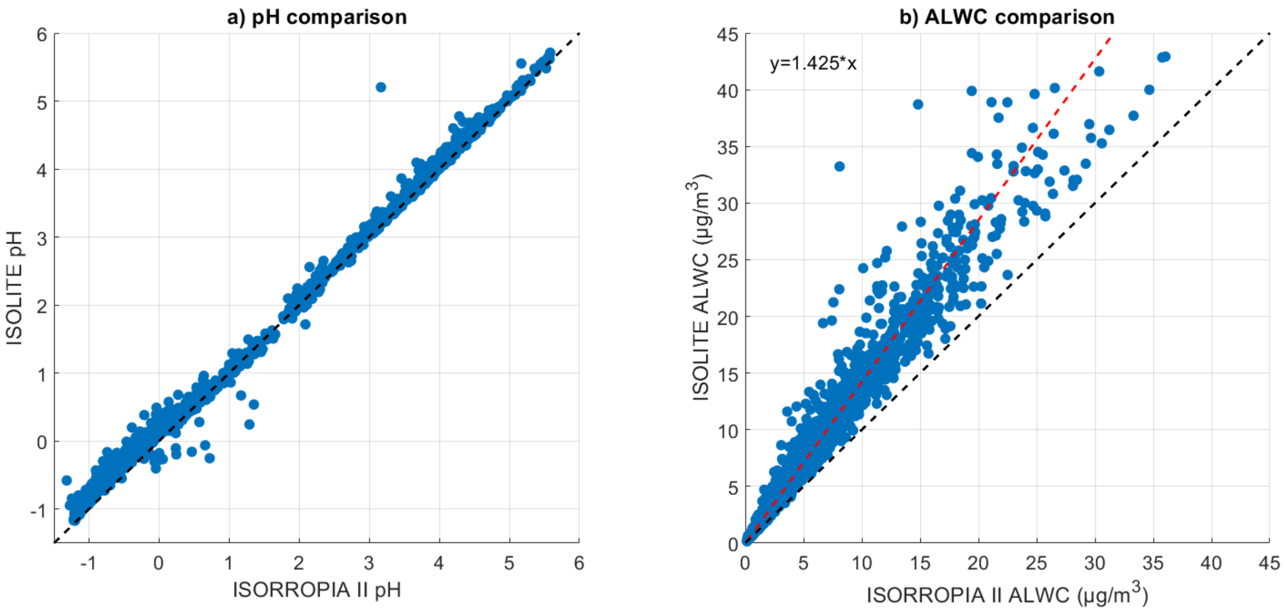

Figure S4. Comparison of outputs from ISORROPIA-Lite and ISORROPIA II. a) Comparison of aerosol liquid water content (ALWC) calculations from ISORROPIA-Lite vs ISORROPIA II. Black line is 1:1 line. Nearly all pH points are on or slightly above the 1:1 line, indicating a very small increase in pH when organic ALWC is accounted for. b) Comparison of ALWC calculations from ISORROPIA-Lite vs ISORROPIA II.

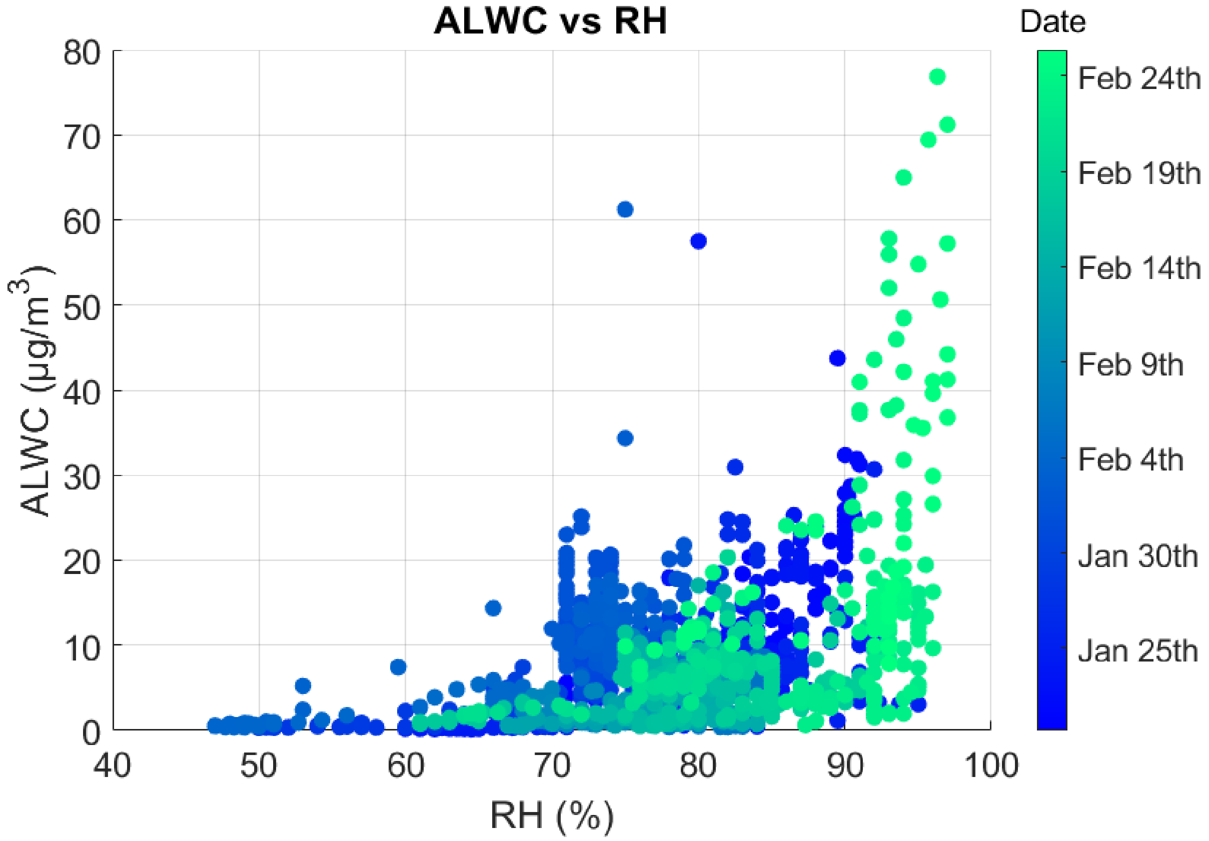

Figure S5. Aerosol liquid water content (ALWC) vs relative humidity (RH), colored by date. ALWC increases dramatically when RH > 90%, which almost exclusively happens during the end of the study period from Feb 23rd to Feb 26th.

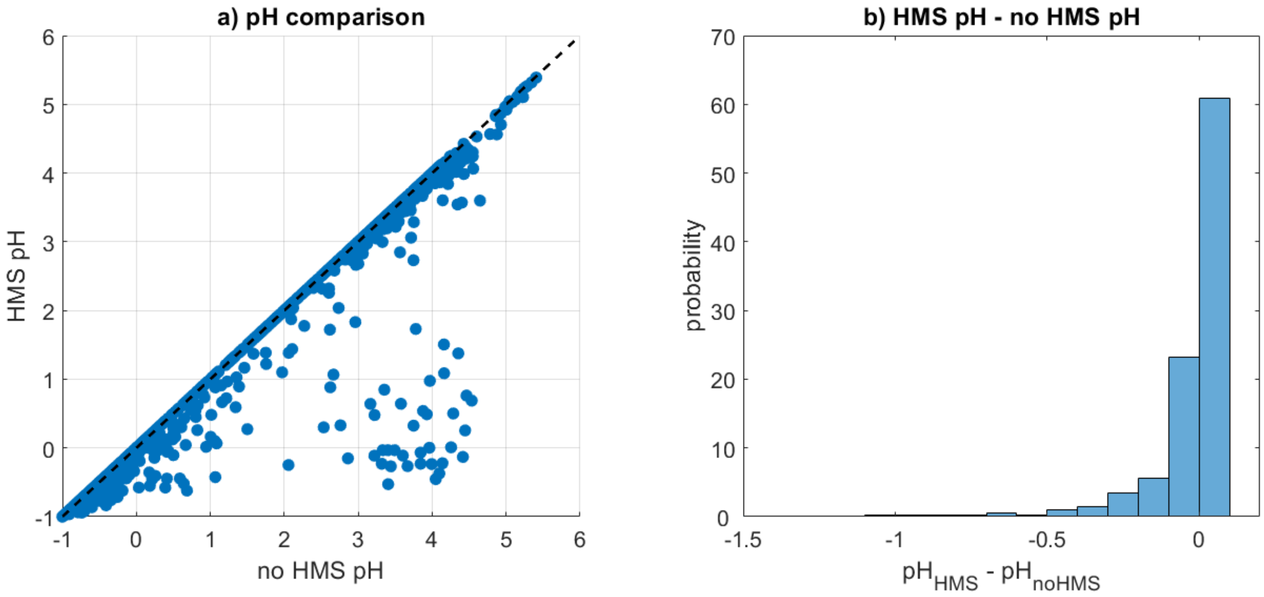

Figure S6. Comparison of pH with and without hydroxymethanesulfonate (HMS) included as a portion of sulfate. a) Scatterplot of pH with and without HMS. Black line is 1:1 line. b) Histogram of pH with HMS minus pH without HMS ($pH_{HMS} - pH_{noHMS}$). The bin width is 0.1 pH.

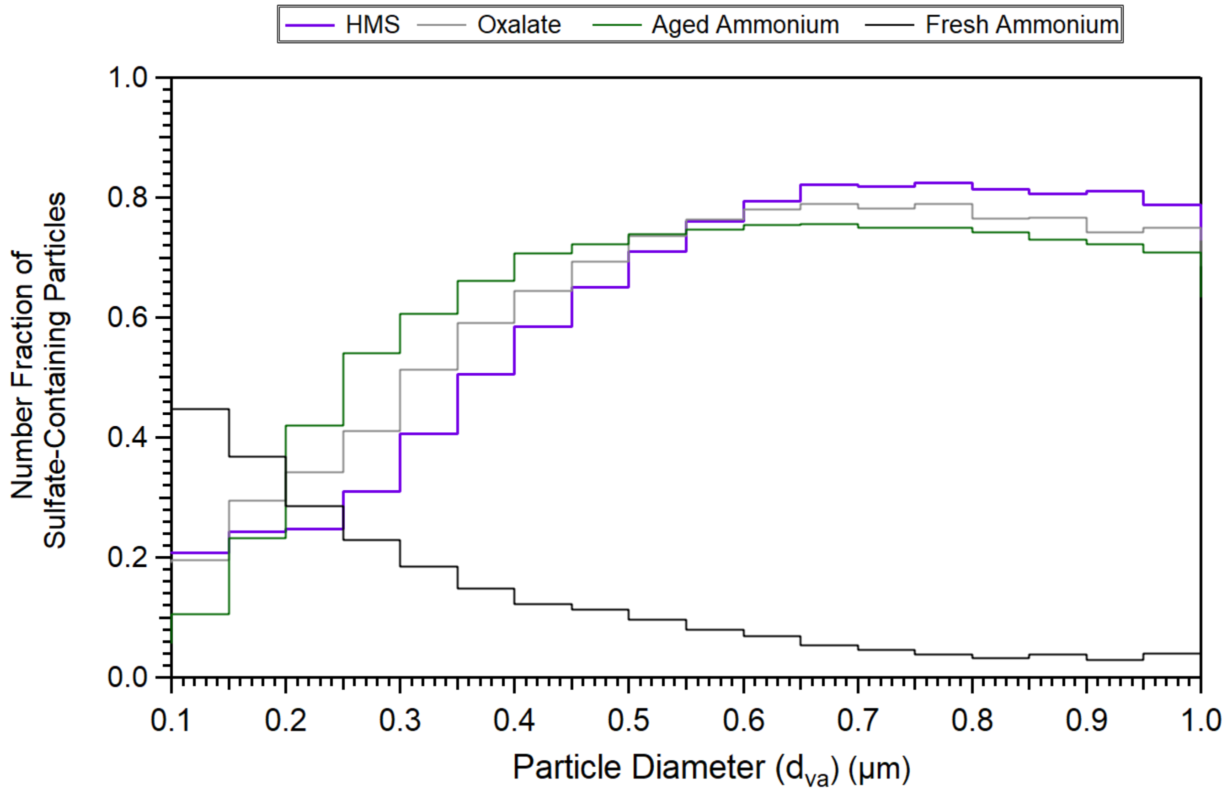

Figure S7. Size-resolved number fractions of individual sulfate-containing particles, measured by the aerosol time-of-flight mass spectrometer (ATOFMS) at the house site, that also contain hydroxymethanesulfonate (HMS), oxalate, "aged" ammonium, and/or "fresh" ammonium, from 0.1 – 1.0 µm in vacuum aerodynamic diameter (d_{va}) with 0.05 µm bin resolution. "Fresh" (recently emitted) ammonium- and sulfate-containing particles are defined as individual particles with mass spectral relative ion peak area ratios of ammonium (*m/z* 18, NH_4^+): elemental carbon (C_n^+) below 0.1; these individual particles have acquired minimal secondary aerosol. As shown, particles classified as containing "fresh" ammonium-containing were most abundant below 0.2 µm, consistent with fresh combustion emissions, accounting for ~40% of measured particles in this size range by number. In contrast, the number fractions of "aged" ammonium, HMS, and oxalate-containing particles are most abundant above ~0.6 µm (~78% of measured individual particles, by number, from 0.6-0.8 µm), consistent with the uptake of water and aqueous-phase chemistry. The number fractions of these particles are comparable at 0.2 µm (25-42%, by number), suggesting that 0.2 µm was the approximate size transition between primarily fresh particles and aged particles. As indicated by the presence of HMS and oxalate, a large number fraction of the particles above 0.2 µm have taken up water, SO_2, HCHO, and NH_3.

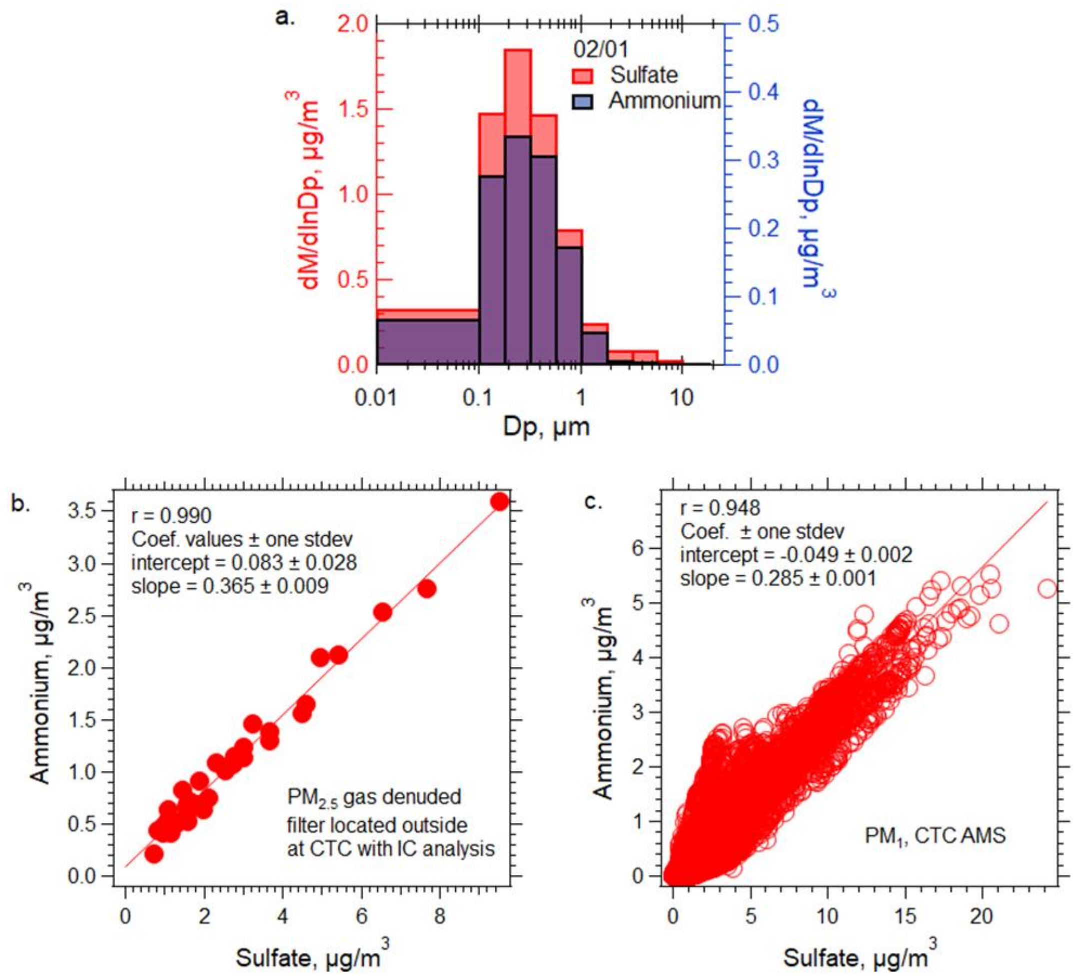

Figure S8. Analysis of sulfate and ammonium size distribution and correlation. a) Sulfate and ammonium mass size distribution during the polluted period. b) $PM_{2.5}$ ammonium vs sulfate mass collected on filters. c) PM_1 ammonium vs sulfate mass measured via aerosol mass spectrometer (AMS). Plots b) and c) use standard regression.

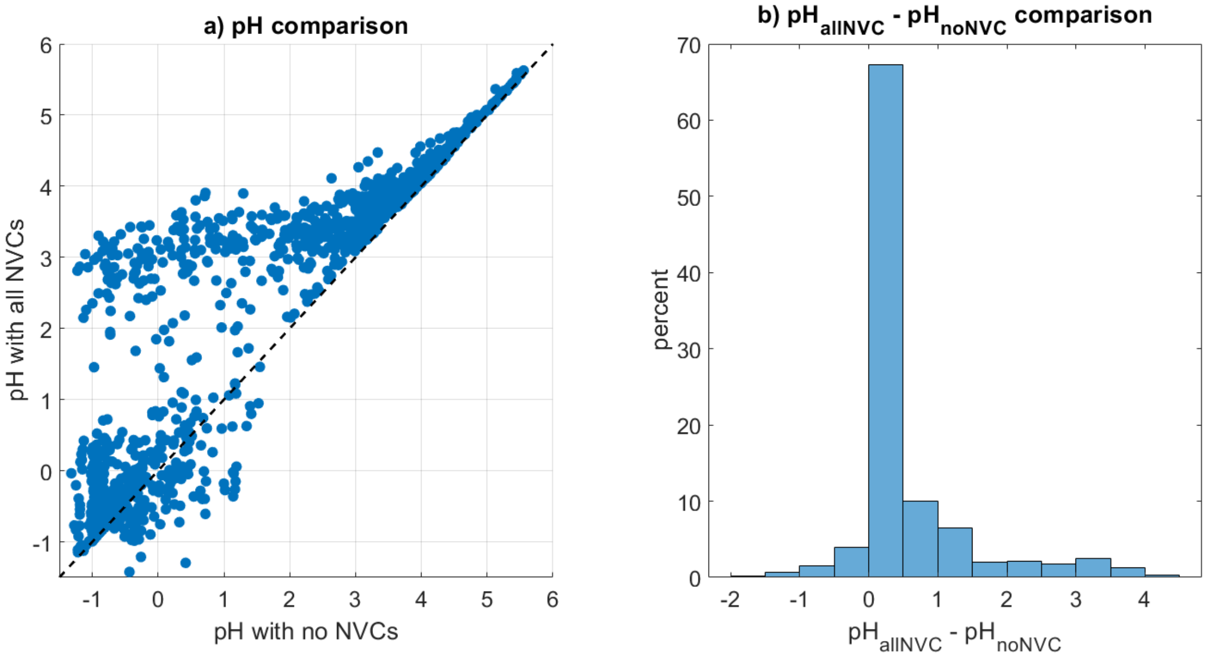

Figure S9. Comparison of pH with and without nonvolatile cations (NVCs). a) Scatterplot of pH with and without NVCs. Black line is 1:1 line. b) Histogram of pH with NVCs minus pH without NVCs (pH_{NVC} – pH_{noNVC}). The bin width is 0.5 pH.

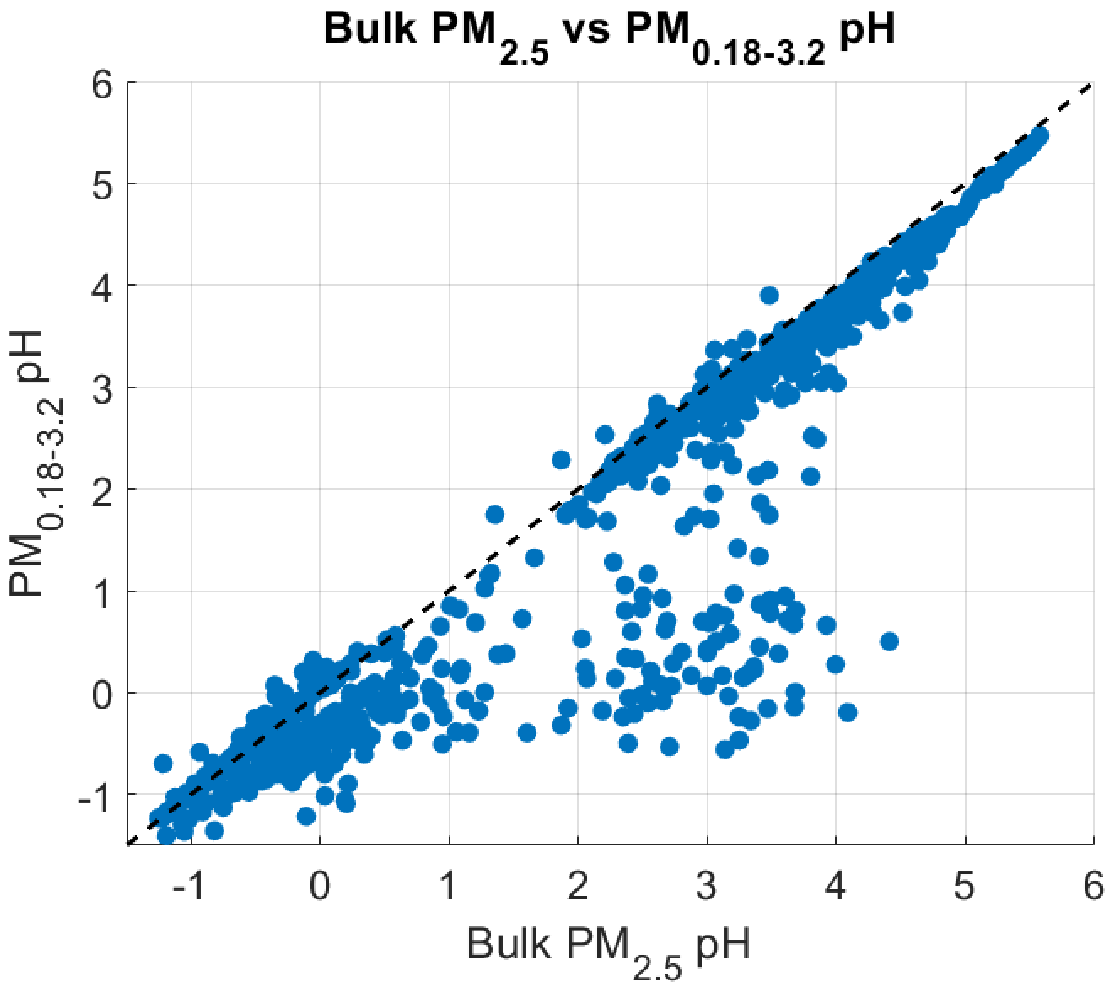

Figure S10. pH calculated based on size-resolved Micro-Orifice Uniform Deposit Impactor data (MOUDI, $PM_{0.18-3.2}$) vs bulk $PM_{2.5}$ pH. "$PM_{0.18-3.2}$" refers to particles less than 0.18 µm being removed from pH calculations. Particles less than 0.2 µm in diameter were shown to be mostly non-deliquesced, so they do not contribute to pH. Using MOUDI data, the mass fractions of sulfate and ammonium from 0.18-3.2 µm were calculated. These mass fractions were applied to PILS sulfate and MC ammonium. The MOUDI size cutoff of 3.2 µm was used because it was the smallest size which included $PM_{2.5}$. Although this includes particles larger than 2.5 µm, MOUDI mass in the size range 1.8-3.2 µm contributed less than 5% of the mass in the total size range 0.18-3.2 µm, so we consider this overestimation to be negligible. Black line is 1:1 line. pH is slightly lower for the $PM_{0.18-3.2}$ size bin due to the increased influence of total nitrate, since nitrate was not scaled down like ammonium and sulfate. Overall, pH results between $PM_{0.18-3.2}$ and bulk $PM_{2.5}$ are consistent.

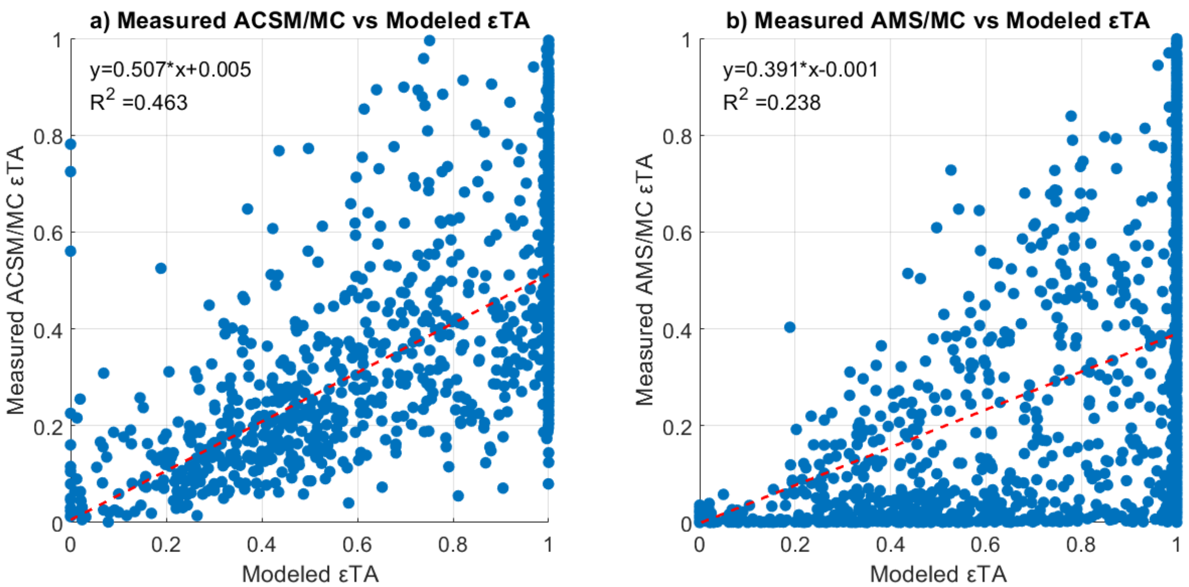

Figure S11. Measured versus modeled ammonia partitioning (εTA) values. a) Aerosol Chemical Speciation Monitor to Mist Chamber (ACSM/MC) vs modeled εTA values. b) Aerosol Mass Spectrometer to Mist Chamber (AMS/MC) vs modeled εTA values. The measured εTA values were calculated by the aerosol-phase species (measured by AMS and ACSM) divided by the total gas + aerosol (MC). York fit applied to both plots.

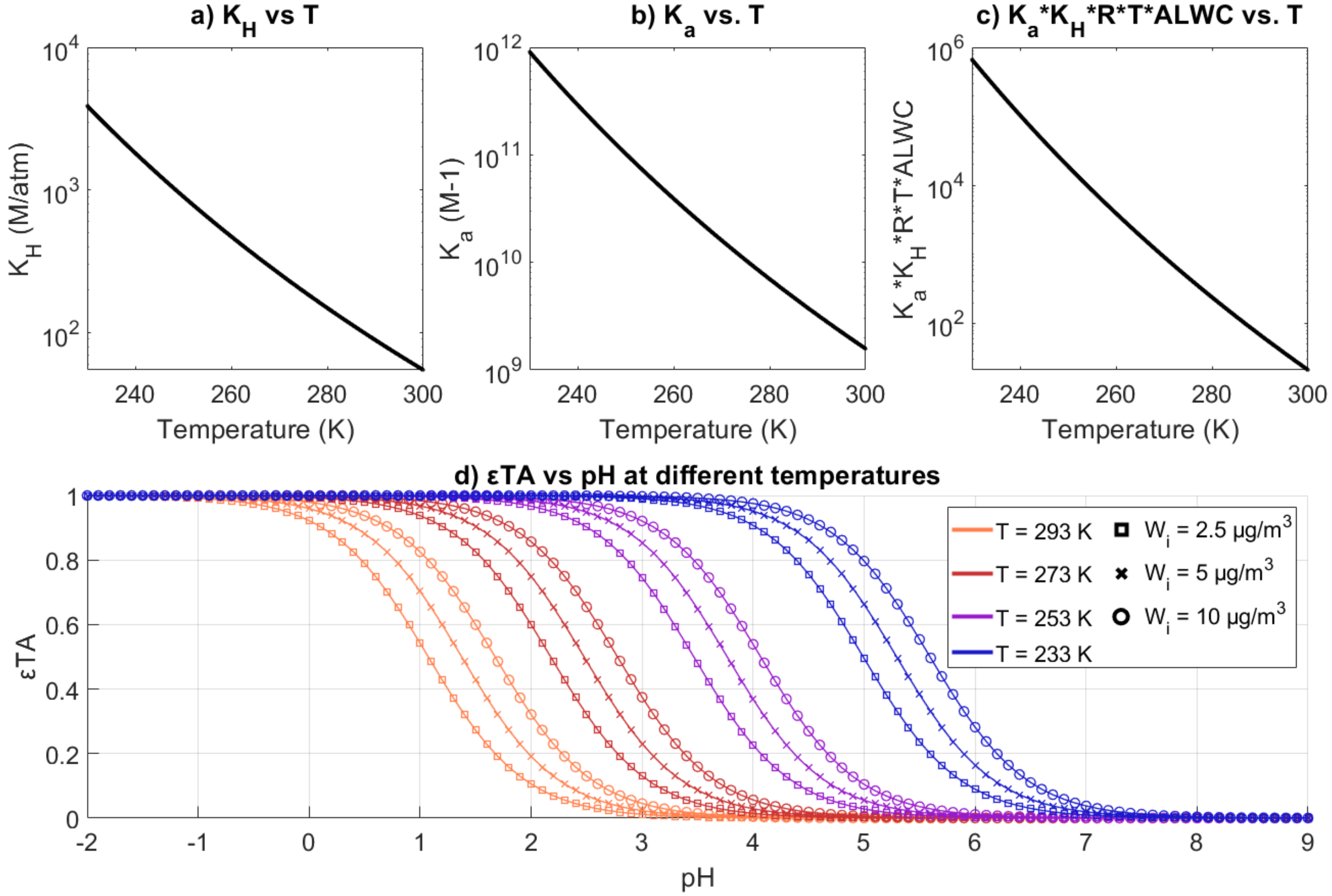

Figure S12. Henry's law (K_H), acid dissociation constant (K_a), and ammonia partitioning (εTA) of ammonia dependence on temperature. a) K_H of ammonia vs temperature, b) K_a of ammonia vs temperature, c) combined effect of plot a and b, and d) εTA vs pH at different temperatures and aerosol liquid water content (ALWC). Note that the y-axis in plots a-c is logarithmic.

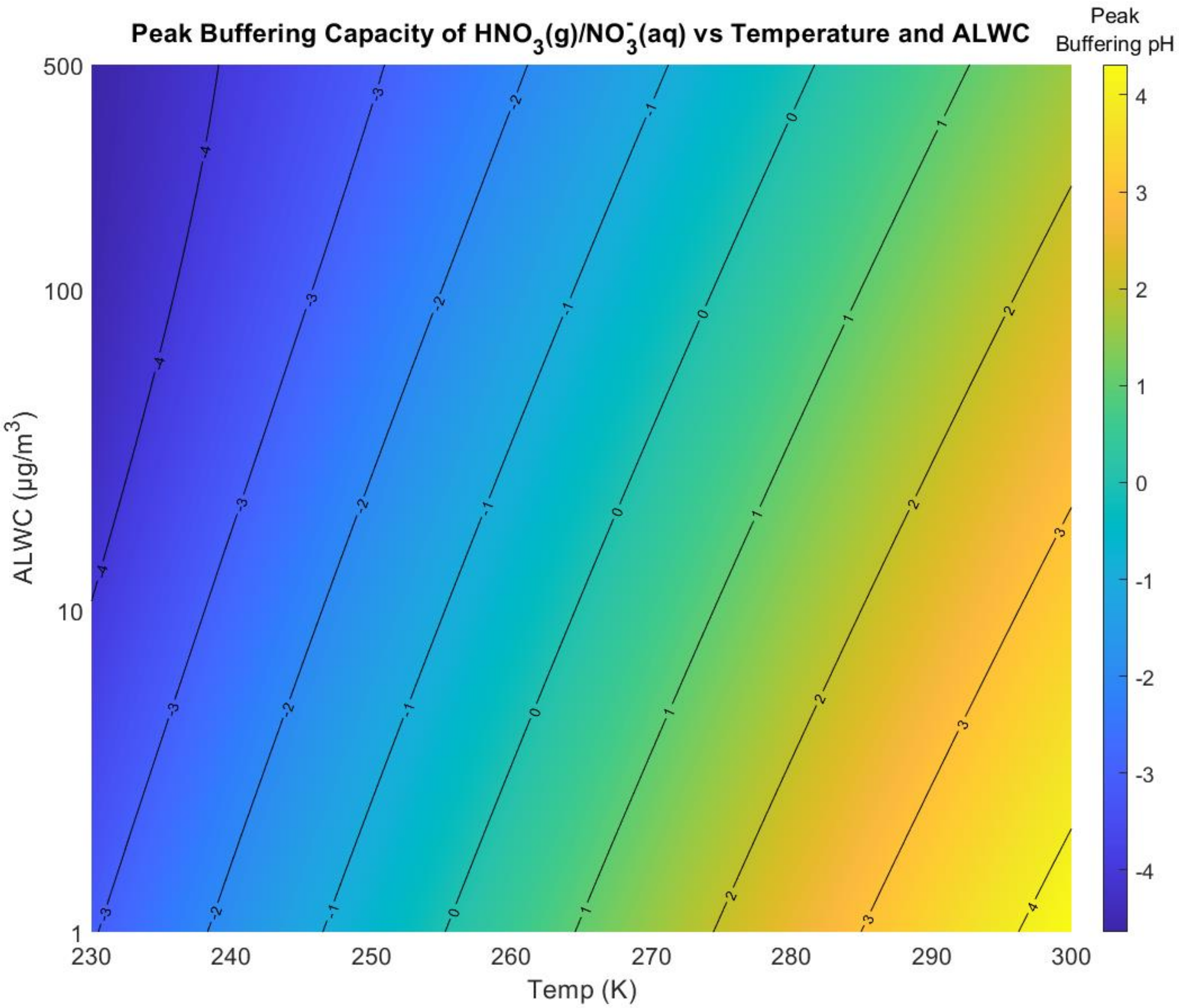

Figure S13. HNO_3(g)/NO_3^-(aq) peak buffering vs temperature and aerosol liquid water content (ALWC).

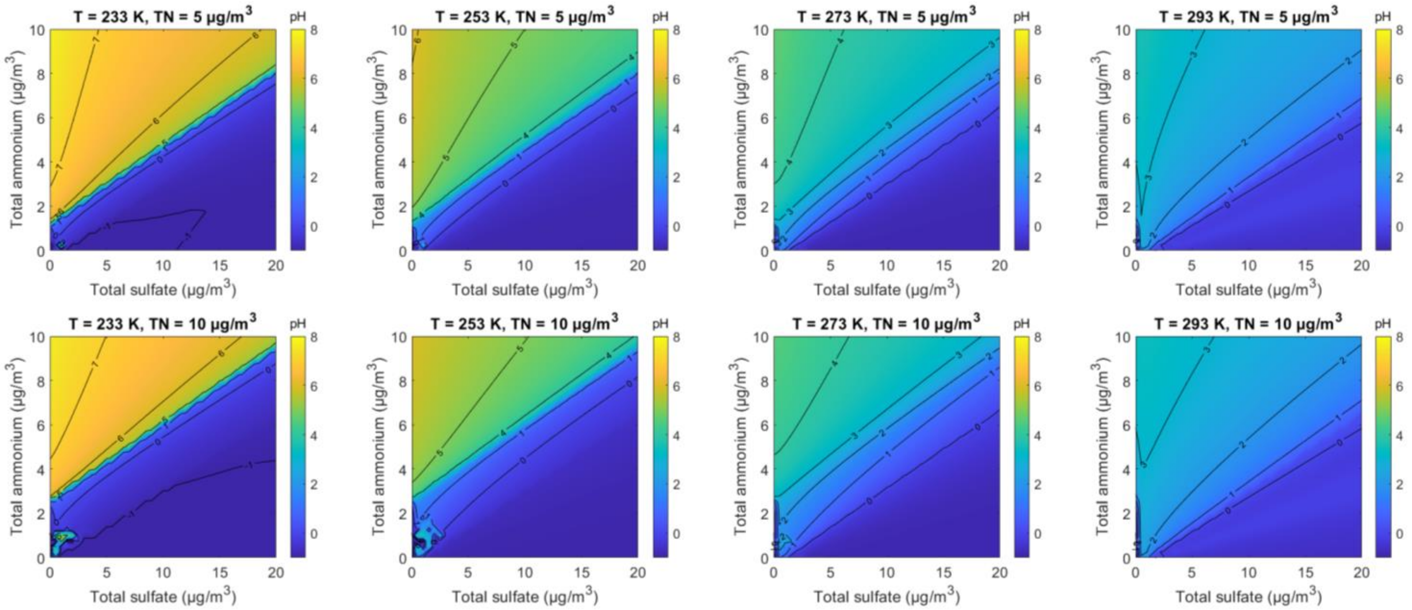

Figure S14. Plots of pH dependence on total ammonium and total sulfate at varying temperature and total nitrate concentration. For variables like chloride and nonvolatile cations, the mean campaign values were used and held constant. Nitrate increases the amount of total ammonium needed to neutralize the aerosol, but otherwise results are similar.

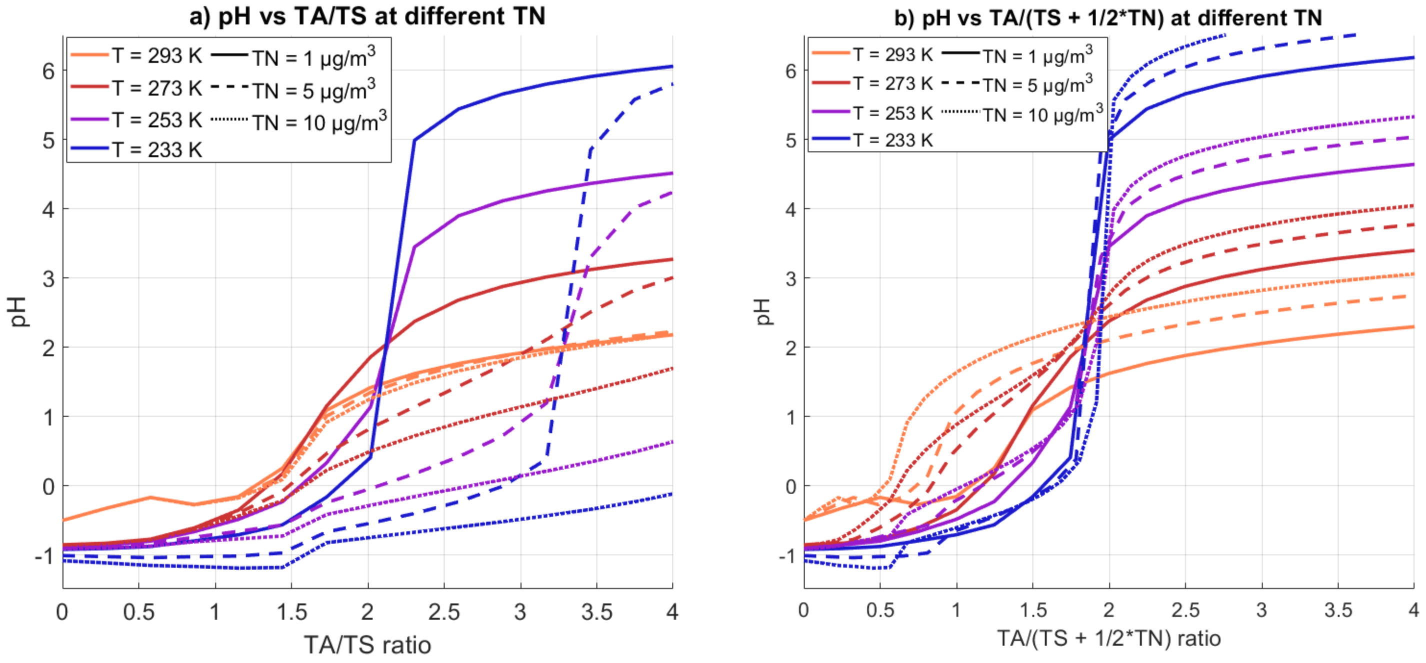

Figure S15. Sensitivity of fine particle pH to total ammonium (TA), total sulfate (TS) and total nitrate (TN) as a function of temperature. a) pH vs TA/TS ratio at different levels of nitrate and b) pH vs TA/(TS + 1/2*TN) ratio at different levels of nitrate. 1/2*TN is used to account for the -2 charge of sulfate and -1 charge of nitrate. pH was calculated in ISORROPIA II at a constant level of sulfate (5 μg/m^3) and variable TA.

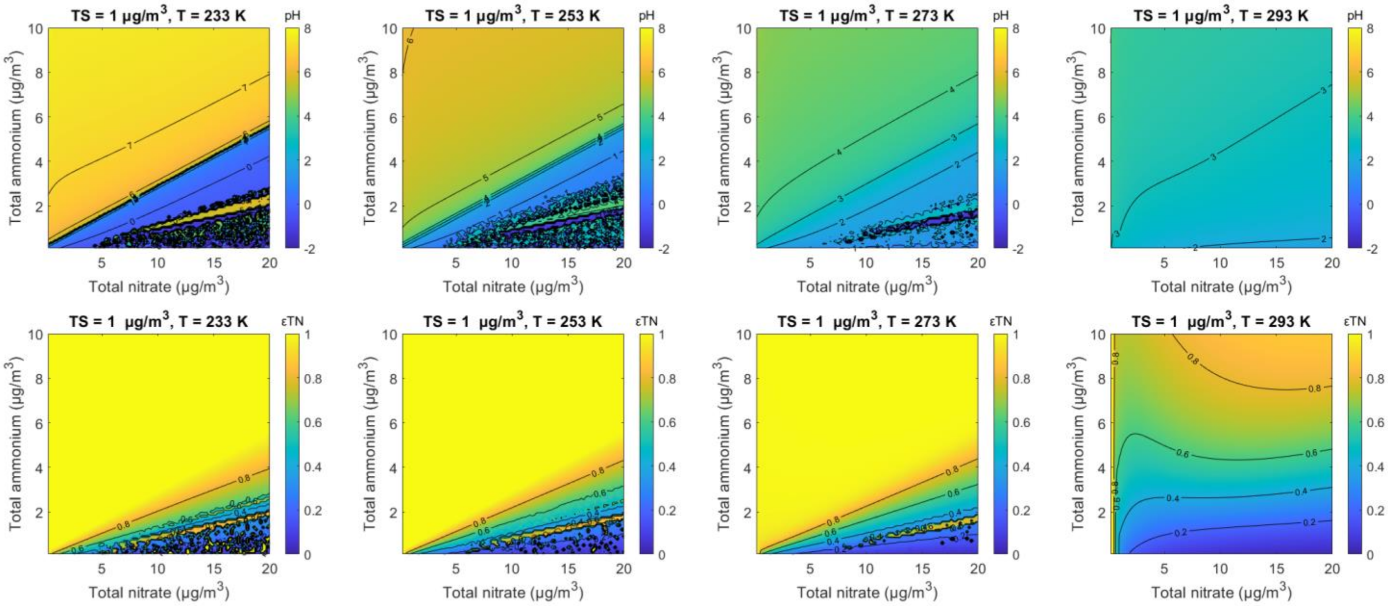

Figure S16. pH and nitric acid partitioning (εTN) dependence on total ammonium to total nitrate ratio (TA/TN) and temperature. Top row shows the how pH changes with TA, TN, and temperature. Bottom row shows how εTN changes with TA, TN, and temperature. Total sulfate (TS) is held constant at 1 μg/m^3.

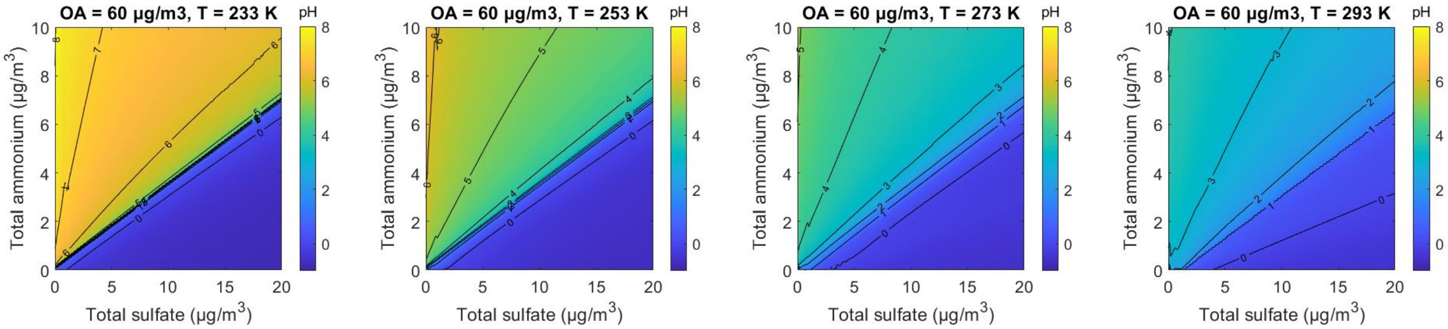

Figure S17. Plots of pH dependence on total ammonium and total sulfate at varying temperature and enhanced organic aerosols (OA) concentration. For variables like total nitrate, chloride, and nonvolatile cations, the mean campaign values were used and held constant. Organic aerosol liquid water content very slightly increases pH.

Table S1. Comparison of global pH estimations.

**Location**	**Season**	**pH**	**Reference**
Southeast US	Summer	0.5-1.5	(27, *23*)
North China Plain	Winter (severe haze period)	5-6	(*21*)
Pasadena, CA, US	Summer	1.5-2.5	(28)
Tianjin, China	Winter	4.2	(*67*, *68*)
Cabauw, Netherlands	Summer	2.8-3.8	(*26*)
	Winter	3.5-4.3	
Crete, Greece	Summer-Fall	0-2.5	(*68*, *38*)
	Yearly Average	2.2-3.4	
Canada	Summer	1.5	(*24*)
	Winter	3.5	

Table S2. Campaign-averaged nonvolatile cation (NVC) measurements for total soluble particles (TSP) and $PM_{2.5}$.

	Na+	K+	Ca+	Mg+	Sum
TSP NVC mass (μg/m^3)	0.2	0.15	0.23	0.03	0.61
Fraction of total NVC mass	32.8%	24.6%	37.7%	4.9%	
$PM_{2.5}$ NVC mass (μg/m^3)	0.06	0.10	0.025	0.023	0.208
Fraction of $PM_{2.5}$ NVC mass	28.8%	48.1%	12.0%	11.1%	

Table S3. **Mean and mass fraction values for aerosol mass spectrometer (AMS), aerosol chemical speciation monitor (ACSM), particle into liquid sampler (PILS), and NCore measurements.** Total $PM_{2.5}$ from NCore was analyzed using a beta attenuation monitor (BAM); NCore sulfate, nitrate, and ammonium were collected on nylon filters and analyzed by IC. Species used in ISORROPIA II and ISORROPIA-Lite calculations are in bold.

		Organic aerosols	Sulfate	Nitrate	Ammonium	Chloride
AMS PM_1 (CTC site)	mass fraction	67.5%	21.6%	6.1%	3.1%	1.7%
	mean (μg/m^3)	6.1	1.7	0.49	0.43	0.11
ACSM $PM_{2.5}$ (NCore site)	mass fraction	71.8%	14.0%	9.0%	5.4%	0.0%
	mean (μg/m^3)	**6.4**	1.3	0.73	0.49	0
PILS $PM_{2.5}$ (CTC site)	mean (μg/m^3)	N/A	**2.7**	**0.9**	**1.4***	0.16
NCore $PM_{2.5}$ (NCore Site)	mass fraction	N/A	20.6%	8.2%	6.8%	1.1%
	mean (μg/m^3)	3.618**	2.738	1.089	0.904	0.152

*Mist chamber (MC) total ammonium (gas phase + aerosol phase).

**Organic carbon (OC), not organic aerosols (OA).

REFERENCES AND NOTES

1. J. Schmale, S. R. Arnold, K. S. Law, T. Thorp, S. Anenberg, W. R. Simpson, J. Mao, K. A. Pratt, Local arctic air pollution: A neglected but serious problem. *Earth's Future* **6**, 1385–1412 (2018).

2. J. K. Kodros, D. K. Papanastasiou, M. Paglione, M. Masiol, S. Squizzato, K. Florou, K. Skyllakou, C. Kaltsonoudis, A. Nenes, S. N. Pandis, Rapid dark aging of biomass burning as an overlooked source of oxidized organic aerosol. *Proc. Natl. Acad. Sci. U.S.A.* **117**, 33028–33033 (2020).

3. J. Xu, D. Imre, R. McGraw, I. Tang, Ammonium sulfate: Equilibrium and metastability phase diagrams from 40 to −50 °C. *J. Phys. Chem. B* **102**, 7462–7469 (1998).

4. T. Koop, B. Luo, A. Tsias, T. Peter, Water activity as the determinant for homogeneous ice nucleation in aqueous solutions. *Nature* **406**, 611–614 (2000).

5. J. H. Seinfeld, S. N. Pandis, *Atmospheric Chemistry and Physics: From Air Pollution to Climate Change* (John Wiley & Sons, 2016).

6. T. Koop, J. Bookhold, M. Shiraiwa, U. Pöschl, Glass transition and phase state of organic compounds: Dependency on molecular properties and implications for secondary organic aerosols in the atmosphere. *Phys. Chem. Chem. Phys.* **13**, 19238 (2011).

7. M. Cesler-Maloney, W. R. Simpson, T. Miles, J. Mao, K. S. Law, T. J. Roberts, Differences in ozone and particulate matter between ground level and 20 m aloft are frequent during wintertime surface-based temperature inversions in Fairbanks, Alaska. *J. Geophys. Res.* **127**, e2021JD036215 (2022).

8. K. C. Nattinger, "Temporal and spatial trends of fine particulate matter composition in Fairbanks, Alaska," thesis, University of Alaska, Fairbanks, AK (2016).

9. J. R. Campbell, M. Battaglia, K. Dingilian, M. Cesler-Maloney, J. M. St Clair, T. F. Hanisco, E. Robinson, P. DeCarlo, W. Simpson, A. Nenes, R. J. Weber, J. Mao, Source and chemistry of hydroxymethanesulfonate (HMS) in Fairbanks, Alaska. *Environ. Sci. Technol.*, **56**, 7657–7667 (2022).

10. R. A. Kotchenruther, Source apportionment of $PM_{2.5}$ at multiple Northwest U.S. sites: Assessing regional winter wood smoke impacts from residential wood combustion. *Atmos. Environ.* **142**, 210–219 (2016).

11. Y. Wang, P. K. Hopke, Is Alaska truly the great escape from air pollution? - Long term source apportionment of fine particulate matter in Fairbanks, Alaska, *Aerosol Air Qual. Res.* **14**, 1875–1882 (2014).

12. T. Ward, B. Trost, J. Conner, J. Flanagan, R. K. M. Jayanty, Source apportionment of $PM_{2.5}$ in a Subarctic Airshed - Fairbanks, Alaska. *Aerosol Air Qual. Res.* **12**, 536–543 (2012).

13. A. Moon, U. Jongebloed, K. K. Dingilian, A. J. Schauer, Y.-C. Chan, M. Cesler-Maloney, W. R. Simpson, R. J. Weber, L. Tsiang, F. Yazbeck, S. Zhai, A. Wedum, A. J. Turner, S. Albertin, S. Bekki, J. Savarino, K. Gribanov, K. A. Pratt, E. J. Costa, C. Anastasio, M. O. Sunday, L. M. D. Heinlein, J. Mao, B. Alexander, Primary sulfate is the dominant source of particulate sulfate during winter in Fairbanks, Alaska. *ACS EST Air* **1**, 139–149 (2024).

14. S. D. Boyce, M. R. Hoffmann, Kinetics and mechanism of the formation of hydroxymethanesulfonic acid at low pH. *J. Phys. Chem.* **88**, 4740–4746 (1984).

15. J. W. Munger, C. Tiller, M. R. Hoffmann, Identification of hydroxymethanesulfonate in fog water. *Science* **231**, 247–249 (1986).

16. J. M. Moch, E. Dovrou, L. J. Mickley, F. N. Keutsch, Y. Cheng, D. J. Jacob, J. Jiang, M. Li, J. W. Munger, X. Qiao, Q. Zhang, Contribution of hydroxymethane sulfonate to ambient particulate matter: A potential explanation for high particulate sulfur during severe winter haze in Beijing. *Geophys. Res. Lett.* **45**, 11969–11979 (2018).

17. T. Ma, H. Furutani, F. Duan, T. Kimoto, J. Jiang, Q. Zhang, X. Xu, Y. Wang, J. Gao, G. Geng, M. Li, S. Song, Y. Ma, F. Che, J. Wang, L. Zhu, T. Huang, M. Toyoda, K. He, Contribution of hydroxymethanesulfonate (HMS) to severe winter haze in the North China Plain. *Atmos. Chem. Phys.* **20**, 5887–5897 (2020).

18. S. Song, M. Gao, W. Xu, Y. Sun, D. R. Worsnop, J. T. Jayne, Y. Zhang, L. Zhu, M. Li, Z. Zhou, C. Cheng, Y. Lv, Y. Wang, W. Peng, X. Xu, N. Lin, Y. Wang, S. Wang, J. W. Munger, D. J. Jacob, M. B. McElroy, Possible heterogeneous chemistry of hydroxymethanesulfonate (HMS) in northern China winter haze. *Atmos. Chem. Phys.* **19**, 1357–1371 (2019).

19. H. Wang, J. Li, T. Wu, T. Ma, L. Wei, H. Zhang, X. Yang, J. W. Munger, F.-K. Duan, Y. Zhang, Y. Feng, Q. Zhang, Y. Sun, P. Fu, M. B. McElroy, S. Song, Model simulations and predictions of Hydroxymethanesulfonate (HMS) in the Beijing-Tianjin-Hebei region, China: Roles of aqueous aerosols and atmospheric acidity. *Environ. Sci. Technol.* **58**, 1589–1600 (2024).

20. H. Zhang, Y. Xu, L. Jia, Hydroxymethanesulfonate formation as a significant pathway of transformation of SO2. *Atmos. Environ.* **294**, 119474 (2023).

21. L. R. Martin, M. P. Easton, J. W. Foster, M. W. Hill, Oxidation of hydroxymethanesulfonic acid by Fenton's reagent. *Atmos. Environ.* **23**, 563–568 (1989).

22. J. M. Moch, E. Dovrou, L. J. Mickley, F. N. Keutsch, Z. Liu, Y. Wang, T. L. Dombek, M. Kuwata, S. H. Budisulistiorini, L. Yang, S. Decesari, M. Paglione, B. Alexander, J. Shao, J. W. Munger, D. J. Jacob, Global importance of hydroxymethanesulfonate in ambient particulate matter: Implications for air quality. *J. Geophys. Res. Atmos.* **125**, e2020JD032706 (2020).

23. H. O. T. Pye, A. Nenes, B. Alexander, A. P. Ault, M. C. Barth, S. L. Clegg, J. L. Collett Jr., K. M. Fahey, C. J. Hennigan, H. Herrmann, M. Kanakidou, J. T. Kelly, I.-T. Ku, V. F. McNeill, N. Riemer, T. Schaefer, G. Shi, A. Tilgner, J. T. Walker, T. Wang, R. Weber, J. Xing, R. A. Zaveri, A. Zuend, The acidity of atmospheric particles and clouds. *Atmos. Chem. Phys.* **20**, 4809–4888 (2020).

24. Y. Cheng, G. Zheng, C. Wei, Q. Mu, B. Zheng, Z. Wang, M. Gao, Q. Zhang, K. He, G. Carmichael, U. Pöschl, H. Su, Reactive nitrogen chemistry in aerosol water as a source of sulfate during haze events in China. *Sci. Adv.* **2**, e1601530 (2016).

25. T. Liu, J. P. D. Abbatt, Oxidation of sulfur dioxide by nitrogen dioxide accelerated at the interface of deliquesced aerosol particles. *Nat. Chem.* **13**, 1173–1177 (2021).

26. H. Guo, L. Xu, A. Bougiatioti, K. M. Cerully, S. L. Capps, J. R. J. Hite Jr, A. G. Carlton, S.-H. Lee, M. H. Bergin, N. L. Ng, A. Nenes, R. J. Weber, Fine-particle water and pH in the southeastern United States. *Atmos. Chem. Phys.* **15**, 5211–5228 (2015).

27. Y. Tao, J. G. Murphy, The sensitivity of $PM_{2.5}$ acidity to meteorological parameters and chemical composition changes: 10-year records from six Canadian monitoring sites. *Atmos. Chem. Phys.* **19**, 9309–9320 (2019).

28. J. P. S. Wong, Y. Yang, T. Fang, J. A. Mulholland, A. G. Russell, S. Ebelt, A. Nenes, R. J. Weber, Fine particle iron in soils and road dust is modulated by coal-fired power plant sulfur. *Environ. Sci. Technol.* **54**, 7088–7096 (2020).

29. H. Guo, R. Otjes, P. Schlag, A. Kiendler-Scharr, A. Nenes, R. J. Weber, Effectiveness of ammonia reduction on control of fine particle nitrate. *Atmos. Chem. Phys.* **18**, 12241–12256 (2018).

30. R. J. Weber, H. Guo, A. G. Russell, A. Nenes, High aerosol acidity despite declining atmospheric sulfate concentrations over the past 15 years. *Nat. Geosci.* **9**, 282–285 (2016).

31. H. Guo, J. Liu, K. D. Froyd, J. M. Roberts, P. R. Veres, P. L. Hayes, J. L. Jimenez, A. Nenes, R. J. Weber, Fine particle pH and gas–particle phase partitioning of inorganic species in Pasadena, California, during the 2010 CalNex campaign. *Atmos. Chem. Phys.* **17**, 5703–5719 (2017).

32. G. Zheng, H. Su, S. Wang, M. O. Andreae, U. Pöschl, Y. Cheng, Multiphase buffer theory explains contrasts in atmospheric aerosol acidity. *Science* **369**, 1374–1377 (2020).

33. C. G. Schmitt, D. Vas, M. Schnaiter, E. Järvinen, L. Hartl, T. Wong, V. Cassella, M. Stuefer, Microphysical characterization of boundary layer ice particles: Results from a 3-year measurement campaign in interior Alaska. *J. App. Meteorol. Climatol.* **63**, 699–716 (2024).

34. S. Song, T. Ma, Y. Zhang, L. Shen, P. Liu, K. Li, S. Zhai, H. Zheng, M. Gao, J. M. Moch, F. Duan, K. He, M. B. McElroy, Global modeling of heterogeneous hydroxymethanesulfonate chemistry. *Atmoss Chem. Phys.* **21**, 457–481 (2021).

35. C. Chen, Z. Zhang, L. Wei, Y. Qui, W. Xu, S. Song, J. Sun, Z. Li, Y. Chen, N. Ma, W. Xu, X. Pan, P. Fu, Y. Sun, The importance of hydroxymethanesulfonate (HMS) in winter haze episodes in North China Plain. *Environ. Res.* **211**, 113093 (2022).

36. X. Ge, Q. Zhang, Y. Sun, C. R. Ruehl, A. Setyan, Effect of aqueous-phase processing on aerosol chemistry and size distributions in Fresno, California, during wintertime. *Environ. Chem.* **9**, 221 (2012).

37. K. A. Pratt, S. M. Murphy, R. Subramanian, P. J. DeMott, G. L. Kok, T. Campos, D. C. Rogers, A. J. Prenni, A. J. Heymsfield, J. H. Seinfeld, K. A. Prather, Flight-based chemical characterization of biomass burning aerosols within two prescribed burn smoke plumes. *Atmos. Chem. Phys.* **11**, 12549–12565 (2011).

38. L. G. Shields, D. T. Suess, K. A. Prather, Determination of single particle mass spectral signatures from heavy-duty diesel vehicle emissions for $PM_{2.5}$ source apportionment. *Atmos. Environ.* **41**, 3841–3852 (2007).

39. S. M. Toner, L. G. Shields, D. A. Sodeman, K. A. Prather, Using mass spectral source signatures to apportion exhaust particles from gasoline and diesel powered vehicles in a freeway study using UF-ATOFMS. *Atmos. Environ.* **42**, 568–581 (2008).

40. B. Ervens, B. J. Turpin, R. J. Weber, Secondary organic aerosol formation in cloud droplets and aqueous particles (aqSOA): A review of laboratory, field and model studies. *Atmos. Chem. Phys.* **11**, 11069–11102 (2011).

41. S. Myriokefalitakis, K. Tsigaridis, N. Mihalopoulos, J. Sciare, A. Nenes, K. Kawamura, A. Segers, M. Kanakidou, In-cloud oxalate formation in the global troposphere: A 3-D modeling study. *Atmos. Chem. Phys.* **11**, 5761–5782 (2011).

42. W. R. Simpson, J. Mao, G. J. Fochesatto, K. S. Law, P. F. DeCarlo, J. Schmale, K. A. Pratt, S. R. Arnold, J. Stutz, J. E. Dibb, J. M. Creamean, R. J. Weber, B. J. Williams, B. Alexander, L. Hu, R. J. Yokelson, M. Shiraiwa, S. Decesari, C. Anastasio, B. D'Anna, R. C. Gilliam, A. Nenes, J. M. St. Clair, B. Trost, J. H. Flynn, J. Savarino, L. D. Conner, N. Kettle, K. M. Heeringa, S. Albertin, A. Baccarini, B. Barret, M. A. Battaglia, S. Bekki, T. J. Brado, N. Brett, D. Brus, J. R. Campbell, M. Cesler-Maloney, S.

Cooperdock, K. C. de Carvalho, H. Delbarre, P. J. DeMott, C. J. S. Dennehy, E. Dieudonné, K. K. Dingilian, A. Donateo, K. M. Doulgeris, K. C. Edwards, K. Fahey, T. Fang, F. Guo, L. M. D. Heinlein, A. L. Holen, D. Huff, A. Ijaz, S. Johnson, S. Kapur, D. T. Ketcherside, E. Levin, E. Lill, A. R. Moon, T. Onishi, G. Pappaccogli, R. Perkins, R. Pohorsky, J.-C. Raut, F. Ravetta, T. Roberts, E. S. Robinson, F. Scoto, V. Selimovic, M. O. Sunday, B. Temime-Roussel, X. Tian, J. Wu, Y. Yang, Overview of the Alaskan Layered Pollution and Chemical Analysis (ALPACA) field experiment. *ACS EST Air* **1**, 200–222 (2024).

43. H. Guo, A. P. Sullivan, P. Campuzano-Jost, J. C. Schroder, F. D. Lopez-Hilfiker, J. E. Dibb, J. L. Jimenez, J. A. Thornton, S. S. Brown, A. Nenes, R. J. Weber, Fine particle pH and the partitioning of nitric acid during winter in the northeastern United States. *J. Geophys. Res. Atmos.* **121**, 10,355–10,376 (2016).

44. W. R. Cofer, V. G. Collins, R. W. Talbot, Improved aqueous scrubber for collection of soluble atmospheric trace gases. *Environ. Sci. Technol.* **19**, 557–560 (1985).

45. L. Zeng, A. P. Sullivan, R. A. Washenfelder, J. Dibb, E. Scheuer, T. L. Campos, J. M. Katich, E. Levin, M. A. Robinson, R. J. Weber, Assessment of online water-soluble brown carbon measuring systems for aircraft sampling. *Atmos. Meas. Tech.* **14**, 6357–6378 (2021).

46. K. A. Pratt, J. E. Mayer, J. C. Holecek, R. C. Moffet, R. O. Sanchez, T. P. Rebotier, H. Furutani, M. Gonin, K. Fuhrer, Y. Su, S. Guazzotti, K. A. Prather, Development and characterization of an aircraft Aerosol time-of-flight mass spectrometer. *Anal. Chem.* **81**, 1792–1800 (2009).

47. M. J. Gunsch, R. M. Kirpes, K. R. Kolesar, T. E. Barrett, S. China, R. J. Sheesley, A. Laskin, A. Wiedensohler, T. Tuch, K. A. Pratt, Contributions of transported Prudhoe Bay oil field emissions to the aerosol population in Utqiaġvik, Alaska, *Atmos. Chem.Phys.* **17**, 10879–10892 (2017).

48. C. A. Noble, K. A. Prather, Real-time measurement of correlated size and composition profiles of individual atmospheric aerosol particles. *Environ. Sci. Technol.* **30**, 2667–2680 (1996).

49. K. R. Neubauer, S. T. Sum, M. V. Johnston, A. S. Wexler, Sulfur speciation in individual aerosol particles. *J. Geophys. Res. Atmos.* **101**, 18701–18707 (1996).

50. P. V. Bhave, J. O. Allen, B. D. Morrical, D. P. Fergenson, G. R. Cass, K. A. Prather, A field-based approach for determining ATOFMS instrument sensitivities to ammonium and nitrate. *Environ. Sci. Technol.* **36**, 4868–4879 (2002).

51. R. C. Sullivan, K. A. Prather, Investigations of the diurnal cycle and mixing state of oxalic acid in individual particles in Asian aerosol outflow. *Environ. Sci. Technol.* **41**, 8062–8069 (2007).

52. J. R. Whiteaker, K. A. Prather, Hydroxymethanesulfonate as a tracer for fog processing of individual aerosol particles. *Atmos. Environ.* **37**, 1033–1043 (2003).

53. J. Liu, M. J. Gunsch, C. E. Moffett, L. Xu, R. El Asmar, Q. Zhang, T. B. Watson, H. M. Allen, J. D. Crounse, J. St. Clair, M. Kim, P. O. Wennberg, R. J. Weber, R. J. Sheesley, K. A. Pratt, Hydroxymethanesulfonate (HMS) formation during summertime fog in an Arctic oil field. *Environ. Sci. Technol. Lett.* **8**, 511–518 (2021).

54. K. Dingilian, E. Hebert, M. Jr. Battaglia, J. R. Campbell, M. Cesler-Maloney, W. Simpson, J. M. St. Clair, J. Dibb, B. Temime-Roussel, B. D'Anna, A. Moon, B. Alexander, Y. Yang, A. Nenes, J. Mao, R. J. Weber, Hydroxymethanesulfonate and sulfur(IV) in Fairbanks winter during the ALPACA Study. *ACS EST Air* **1**, 646–659 (2024).

55. X. Rao, J. L. Jr Collett, Behavior of S(IV) and formaldehyde in a chemically heterogeneous cloud. *Environ. Sci. Technol.* **29**, 1023–1031 (1995).

56. A. M. Middlebrook, R. Bahreini, J. L. Jimenez, M. R. Canagaratna, Evaluation of composition-dependent collection efficiencies for the aerodyne aerosol mass spectrometer using field data. *Aerosol Sci. Tech.* **46**, 258–271 (2012).

57. J. Peck, L. A. Gonzalez, L. R. Williams, W. Xu, P. L. Croteau, M. T. Timko, J. T. Jayne, D. R. Worsnop, R. C. Miake-Lye, K. A. Smith, Development of an aerosol mass spectrometer lens system for PM2.5. *Aerosol Sci. Technol.* **50**, 781–789 (2016).

58. W. Hu, P. Campuzano-Jost, D. A. Day, P. Croteau, M. R. Canagaratna, J. T. Jayne, D. R. Worsnop, J. L. Jimenez, Evaluation of the new capture vaporizer for aerosol mass spectrometers (AMS) through field studies of inorganic species. *Aerosol Sci. Tech.* **51**, 735–754 (2017).

59. T. Joo, Y. Chen, W. Xu, P. Croteau, M. R. Canagaratna, D. Gao, H. Guo, G. Saavedra, S. S. Kim, Y. Sun, R. Weber, J. Jayne, N. L. Ng, Evaluation of a new Aerosol Chemical Speciation Monitor (ACSM) system at an urban site in Atlanta, GA: The use of capture vaporizer and PM2.5 inlet. *ACS Earth Space Chem.* **5** 2565–2576 (2021).

60. C. Fountoukis, A. Nenes, ISORROPIA II: A computationally efficient thermodynamic equilibrium model for K^{+}–Ca^{2+}–Mg^{2+}–NH^{4+}–Na^{+}–SO_4^{2-}–NO_3^{-}–Cl^{-}–H_2O aerosols. *Atmos. Chem. Phys.*, **7** 1893–1939 (2007).

61. S. Kakavas, S. N. Pandis, A. Nenes, ISORROPIA-lite: A comprehensive atmospheric aerosol thermodynamics module for Earth System Models. *Tellus B Chem. Phys. Meteorol.* **74**, 1 (2022).

62. V. A. Marple, K. L. Rubow, S. M. Behm, A microorifice uniform deposit impactor (MOUDI): Description, calibration, and use. *Aerosol Sci. Tech.* **14**, 434–446 (1991).

63. G. Wang, R. Zhang, M. E. Gomez, L. Yang, M. L. Zamora, M. Hu, Y. Lin, J. Peng, S. Guo, J. Meng, J. Li, C. Cheng, T. Hu, Y. Ren, Y. Wang, J. Gao, J. Cao, Z. An, W. Zhou, G. Li, J. Wang, P. Tian, W. Marrero-Ortiz, J. Secrest, Z. Du, J. Zheng, D. Shang, L. Zeng, M. Shao, W. Wang, Y. Huang, Y. Wang, Y. Zhu, Y. Li, J. Hu, B. Pan, L. Cai, Y. Cheng, Y. Ji, F. Zhang, D. Rosenfeld, P. S. Liss, R. A. Duce, C. E. Kolb, M. J. Molina, Persistent sulfate formation from London Fog to Chinese haze. *Proc. Natl. Acad. Sci. U.S.A.* **113**, 13630–13635 (2016).

64. A. Bougiatioti, P. Nikolaou, I. Stavroulas, G. Kouvarakis, R. Weber, A. Nenes, M. Kanakidou, N. Mihalopoulos, Particle water and pH in the eastern Mediterranean: Source variability and implications for nutrient availability. *Atmos. Chem. Phys.* **16**, 4579–4591 (2016).

65. R. Sander, Compilation of Henry’s law constants (version 4.0) for water as solvent *Chem. Phys.* **15**, 4399–4981 (2015).

66. Y. P. Kim, J. H. Seinfeld, P. Saxena, Atmospheric gas-aerosol equilibrium I. thermodynamic model. *Aerosol. Sci. Technol.* **19**, 157–181 (2007).

67. H. L. Wang, L. P. Qiao, S. R. Lou, M. Zhou, A. J. Ding, H. Y. Huang, J. M. Chen, Q. Wang, S. K. Tao, C. H. Chen, L. Li, C. Huang, Chemical composition of $PM_{2.5}$ and meteorological impact among three years in urban Shanghai, China. *J. Clean. Prod.* **112**, 1302–1311 (2016).

68. H. Guo, R. J. Weber, A. Nenes, High levels of ammonia do not raise fine particle pH sufficiently to yield nitrogen oxide-dominated sulfate production. *Sci. Rep.* **7**, 12109 (2017).
